# Predictors of fertility preservation awareness and willingness among college students and the general population in Henan, China: a cross-sectional study

**DOI:** 10.3389/fpubh.2026.1830907

**Published:** 2026-06-03

**Authors:** Lu Wang, Yijun Zhao, Huanhuan Chen, Lei Zhang, Chenchen Cui, Cuilian Zhang, Linlin Liang

**Affiliations:** 1 Reproductive Medicine Center, Henan Provincial People’s Hospital, People's Hospital of Zhengzhou University, Zhengzhou, Henan, China; 2 Henan Joint International Research Laboratory of Reproductive Bioengineering, Zhengzhou, Henan, China; 3 Department of Obstetrics and Gynecology, The Third Affiliated Hospital of Zhengzhou University, Zhengzhou, Henan, China

**Keywords:** China, college students, cross-sectional study, fertility preservation, knowledge score, logistic regression, population attributable fraction, reproductive health literacy

## Abstract

**Background:**

Fertility preservation (FP) enables individuals to maintain future reproductive potential, yet population-level data on FP awareness and willingness remain limited in inland China. Previous studies have been largely descriptive without quantifying the population-level impact of modifiable determinants. This study identified independent predictors of FP awareness and willingness among college students and the general population in Henan Province, central China, and estimated the population attributable fraction (PAF) of key modifiable factors.

**Methods:**

A cross-sectional survey was conducted (May–October 2022) using stratified sampling among college students (*n* = 765) and general population members (*n* = 1,008). Two validated knowledge scoring systems (13-point and 11-point scales) were developed. Multivariable logistic regression and PAF analysis were performed.

**Results:**

Among college students, FP awareness was 21.1% and willingness 65.3%. Knowledge score was the sole independent predictor of FP awareness (aOR = 2.22, 95%CI: 1.92–2.56; PAF = 62.8%). FP willingness was independently associated with medical major (aOR = 1.62, *p* = 0.003), contraceptive use (aOR = 2.06, PAF = 48.2%), and gender (aOR for female = 0.68, *p* = 0.024). In the general population, knowledge score (aOR = 1.86, *p* < 0.001) and medical background (aOR = 1.83, *p* = 0.003) predicted FP awareness. FP willingness was independently associated with marital status (aOR = 2.10, PAF = 39.7%) and knowledge score (aOR = 1.16, *p* < 0.001). Educational attainment was not independently associated with FP willingness after adjustment (aOR = 1.09, *p* = 0.198). Sensitivity analyses using corrected risk ratios confirmed that OR-based PAFs represent upper-bound estimates for common outcomes.

**Conclusion:**

Knowledge is the primary modifiable predictor of FP awareness and willingness with substantial population-level impact. If confirmed in longitudinal studies, knowledge-centered educational strategies prioritizing non-medical populations, unmarried individuals, and rural residents may substantially reduce current awareness and willingness gaps.

## 1 Introduction

Delayed childbearing has become a defining demographic trend in many countries, including China (1). Over the past two decades, the mean age at first birth among Chinese women has risen substantially, from the mid-twenties around the year 2000 to approximately 30 years by 2020 in urban areas, with even higher ages reported in first-tier metropolitan cities such as Shanghai and Beijing (2). This shift is driven by a complex interplay of factors, including rising educational attainment, increasing female labor force participation, evolving gender norms, intensifying economic pressures related to housing and childcare, and the progressive liberalization of family planning policies (3, 4). As a consequence, a growing proportion of individuals and couples are entering their peak reproductive years at a time when age-related fertility decline has become a clinically significant concern, particularly for women, in whom ovarian reserve and oocyte quality diminish markedly after the age of 35 (5, 6).

Fertility preservation (FP) has emerged as an important component of reproductive medicine, encompassing techniques such as oocyte, sperm, embryo, and ovarian tissue cryopreservation (7, 8). Originally developed for patients facing gonadotoxic treatments, FP is now increasingly considered by individuals deferring childbearing for social or economic reasons (9). Advances in vitrification and post-thaw survival rates, together with professional society guidelines recognizing oocyte cryopreservation for non-medical indications (7, 10, 11), have established FP as a mainstream reproductive health option.

However, the potential benefits of FP can only be realized if individuals are aware of its existence, understand its indications and limitations, and are willing to consider it as part of their reproductive planning (12). Evidence from high-income countries indicates that public awareness of FP remains limited, even among populations with relatively high educational attainment and good access to healthcare services (13). Studies conducted in the United States, Europe, and Australia have consistently reported that many reproductive-age adults have little or no knowledge of FP options, and that awareness is strongly influenced by sociodemographic factors such as gender, education level, income, and exposure to health information (14–17). These findings suggest that informational barriers may be as important as financial or logistical barriers in limiting the uptake of FP services.

In China, research on FP awareness and attitudes has begun to accumulate but remains limited in several important respects. First, most existing studies have focused on specific clinical populations, such as oncology patients or individuals attending reproductive medicine clinics, rather than on the general public or young adults who may benefit from early awareness of FP options (18–22). Second, the majority of available population-based studies have been conducted in economically developed coastal cities and first-tier metropolitan areas, leaving a substantial gap in knowledge regarding FP-related awareness and attitudes in inland provinces and regions with less developed healthcare infrastructure (19, 22–24). Third, prior studies have relied predominantly on descriptive statistics and bivariate analyses, such as frequency distributions and chi-square tests, without using multivariable models to adjust for confounding or quantify the independent contribution of modifiable determinants (19–24). To date, limited research in China has integrated multivariable regression with population attributable fraction (PAF) analysis to estimate the population-level impact of modifiable predictors of FP awareness and willingness.

Henan Province represents a particularly informative setting in which to address these gaps. As one of China’s most populous provinces, with over 99 million residents (25), Henan has demographic and socioeconomic characteristics that broadly reflect national trends in urbanization, educational expansion, and fertility transition, while also exhibiting pronounced disparities in healthcare resource allocation between urban centers and rural areas (26–28). The province encompasses a wide spectrum of socioeconomic conditions, ranging from rapidly developing urban cores with access to tertiary reproductive medicine centers to remote rural communities where basic reproductive health services may be limited. This heterogeneity makes Henan an ideal setting for investigating how sociodemographic factors, educational background, and health literacy interact to shape FP awareness and attitudes across diverse population segments. Moreover, as an inland province that has received comparatively little attention in the reproductive health literature, data from Henan can complement and extend findings from studies conducted in China’s more economically developed coastal region (21–23).

To address these gaps, the present study was designed with the following objectives: (1) to assess levels of FP awareness and willingness to use FP services among college students and the general population in Henan Province; (2) to develop and validate knowledge scoring systems that capture both basic reproductive health knowledge and FP-specific knowledge; (3) to identify independent predictors of FP awareness and willingness through multivariable logistic regression, controlling for potential confounders; (4) to quantify the population-level impact of key modifiable determinants using PAF analysis, thereby providing an evidence base for prioritizing public health interventions; and (5) to compare predictor patterns across the college student and general population samples in order to determine whether unified or population-specific intervention strategies are warranted.

The candidate predictors examined in this study — including gender, educational background, place of residence, marital status, knowledge level, and reproductive health behaviors — were selected based on their established associations with reproductive health awareness in previous studies, their potential modifiability through public health interventions, and their relevance to the sociocultural context of inland China where traditional attitudes toward fertility may influence awareness and willingness patterns.

## 2 Methods

### 2.1 Study design and participants

A cross-sectional survey was conducted in Henan Province, China, between May 1 and October 31, 2022, to assess awareness, knowledge, and attitudes toward fertility preservation (FP) among two distinct population groups: college students and members of the general public. The study was designed to capture variation across key sociodemographic subgroups and to reflect both urban and rural contexts within the province.

#### 2.1.1 Sampling strategy

A stratified sampling approach was used to obtain adequate representation across demographic and socioeconomic strata. Stratification factors included type of residence (urban, rural town, village), gender, educational attainment, academic major (for college students), occupational field (for the general population), and indicators of socioeconomic status. Within each stratum, participants were recruited using a combination of institutional and community-based channels.

The dual-mode administration strategy (online and paper-based) may introduce differential participation patterns, as online respondents may differ from paper-based respondents in demographic characteristics and health literacy. The stratified sampling approach was designed to mitigate this by ensuring representation across key demographic strata. However, because a probability sampling frame was not employed, the sample should not be regarded as strictly representative of the entire population of Henan Province, and results should be interpreted accordingly.

#### 2.1.2 Inclusion and exclusion criteria

Eligibility criteria for participation were as follows. For college students: age 18 years or older, current enrollment in a college or university in Henan Province, ability to read and understand Mandarin Chinese, and willingness and capacity to provide verbal informed consent. For members of the general population: age between 18 and 45 years, current residence in Henan Province, ability to read and understand Mandarin Chinese, and willingness and capacity to provide verbal informed consent.

Exclusion criteria for both groups included: failure to meet the age or residence criteria; cognitive impairment or other conditions that precluded provision of informed consent or completion of the questionnaire; incomplete survey responses, defined as more than 20% missing data on key variables; and evidence of duplicate participation, identified through response patterns and, where applicable, repeated contact information.

#### 2.1.3 Sample size determination

**Figure 1:**
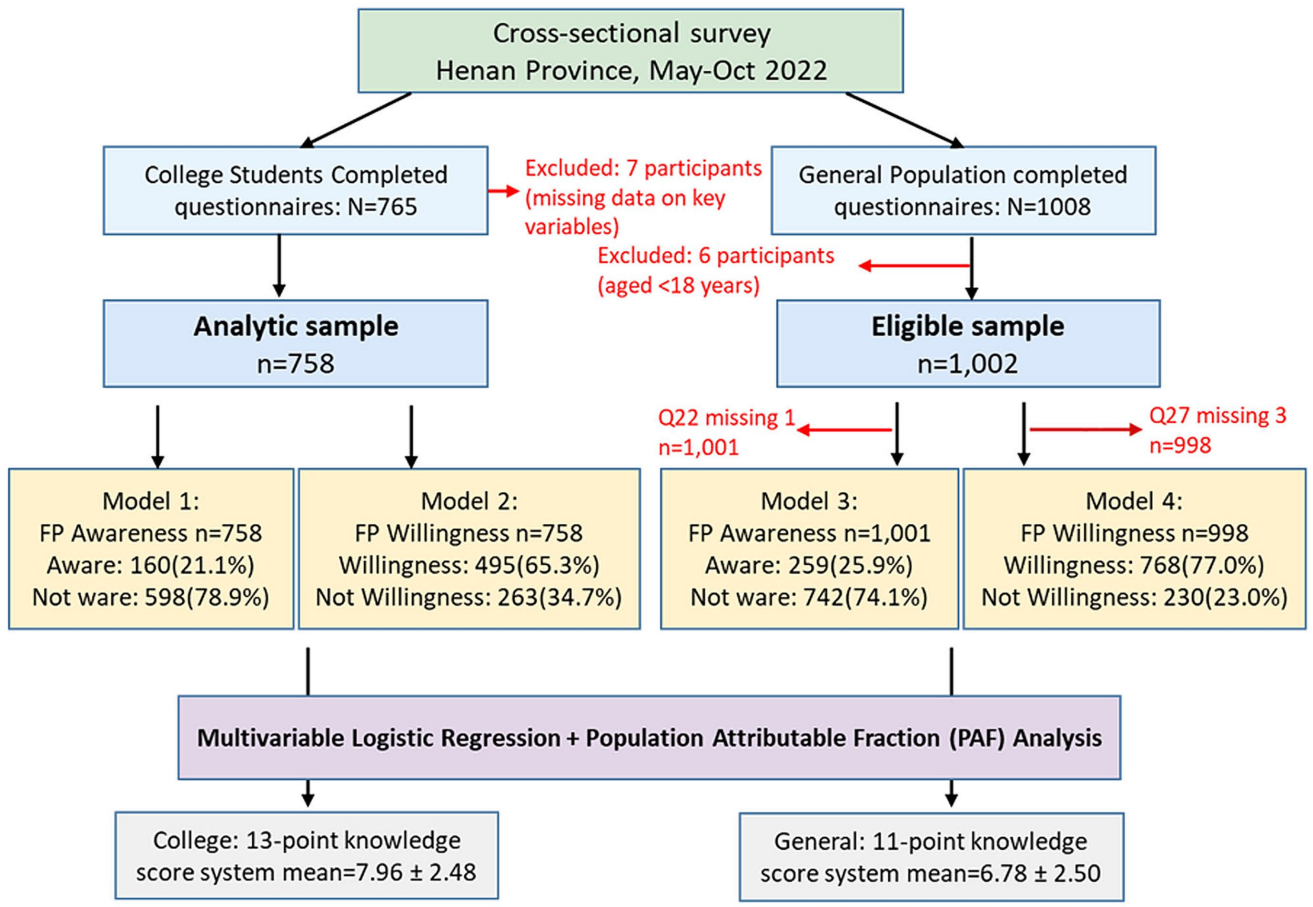
Participant flow diagram. Flow of participants through the study from initial enrollment to final analytic samples. College students: 765 enrolled, 7 excluded for missing data on key variables, yielding *n* = 758 for Models 1 and 2. General population: 1,008 enrolled, 6 excluded for age < 18 years, with additional exclusions for missing outcome data yielding *n* = 1,001 for Model 3 (FP awareness) and *n* = 998 for Model 4 (FP willingness). Knowledge was assessed using a 13-point scale (college students) and an 11-point scale (general population).

Sample size was determined using formal power calculations to ensure adequate statistical power for detecting meaningful differences in FP awareness and willingness between key subgroups. The parameters were set as follows: two-sided significance level (*α*) = 0.05, target statistical power (1 − *β*) = 0.80, and an anticipated medium effect size (Cohen’s d = 0.50), informed by effect sizes reported in previous studies on reproductive health knowledge and attitudes in Chinese populations. The resulting minimum total sample size required for the combined college student and general population samples was 1,773 participants. In addition, the sampling plan aimed to include at least 65 to 70 participants in each key subgroup defined by the intersection of major stratification variables (e.g., gender × residence type × academic major), in order to allow for stable estimation and meaningful subgroup comparisons. The final achieved sample comprised 765 college students and 1,008 members of the general population, yielding a total of 1,773 participants, which met the predetermined sample size requirement. After application of exclusion criteria, the analytic samples comprised 758 college students and up to 1,001 general population participants (see §2.4.5 and Figure 1 for details).

#### 2.1.4 Data collection procedures

Data were collected using a dual-mode administration strategy to maximize participation and ensure inclusion of individuals with varying levels of internet access. The online survey was implemented through Wenjuanxing (www.wjx.cn), a widely used web-based survey platform in China with established data security protocols, and was disseminated via institutional mailing lists, social media groups, and university communication channels. In parallel, paper-based questionnaires were distributed at selected colleges, universities, and community settings—including residential neighborhoods, workplaces, and primary healthcare facilities—to reach individuals with limited internet access or those who preferred a paper format. All participants completed a structured, self-administered questionnaire in Mandarin Chinese, with an estimated completion time of 15–20 min. The questionnaire was anonymous, and no personally identifiable information was collected, stored, or linked to survey responses at any stage of the study.

### 2.2 Survey instrument

#### 2.2.1 Questionnaire development and pilot testing

The survey instrument was a structured questionnaire developed specifically for this study, informed by a review of existing instruments used in reproductive health awareness research and adapted to the Chinese sociocultural context. The questionnaire was pilot-tested among a convenience sample of 758 individuals to evaluate item clarity, response patterns, internal consistency, and completion time. Feedback from the pilot phase led to minor revisions in the wording and ordering of selected items to improve comprehensibility and reduce ambiguity. In addition, the questionnaire content was reviewed by three reproductive medicine specialists at Henan Provincial People’s Hospital for clinical accuracy and relevance. Item-level feedback from both the expert review and pilot testing informed the final selection and wording of knowledge items. The final questionnaire was organized into several sections covering sociodemographic characteristics, reproductive health and FP-related knowledge, FP attitudes and decision-making factors, and FP-specific knowledge, with certain sections administered to specific population groups (college students only, general population only, or both).

#### 2.2.2 Questionnaire structure

The final questionnaire was organized into several sections, with certain sections administered to specific population groups:

Section A: Sociodemographic characteristics (all participants). This section collected information on age, gender, educational level, place of residence (urban area, rural town, rural village), marital status, parental status, and sibling status (only child vs. having siblings). For college students, additional items assessed academic major (medical, science, or liberal arts), current degree program (undergraduate, postgraduate), relationship status (single, in a relationship, married), history of premarital sexual activity, and contraceptive use. For members of the general population, additional items assessed monthly household income, occupational field, and number of children.
Section B: Reproductive health knowledge and FP-related knowledge (college students). This section included items assessing understanding of female reproductive physiology and the fertile window within the menstrual cycle, knowledge of the optimal age for childbearing in women, awareness of the age range at which female fertility declines significantly, perceptions of whether medical interventions can fully restore fertility once it has declined, awareness of trends in infertility prevalence, knowledge of factors that contribute to fertility decline (including cancer treatment, autoimmune disease, advanced age, environmental pollution, unhealthy diet, high-risk occupations, and reproductive system diseases), familiarity with the concept of FP, understanding of the indications for FP, knowledge of specific FP methods (such as oocyte, sperm, embryo, and ovarian tissue cryopreservation), and attitudes toward assisted reproductive technologies, adoption, and voluntary childlessness as responses to potential infertility.
Section C: FP attitudes and decision-making factors (general population including a subset of college students). This section evaluated willingness to use FP services under different scenarios, perceived benefits and concerns related to FP, and factors influencing FP decision-making (including costs, success rates, ethical considerations, and family or partner opinions). Prior FP knowledge was additionally measured using a self-rated 10-point Likert-type scale, with higher scores indicating greater self-perceived understanding of FP.
Section D: FP-specific knowledge (general population). This section included items assessing awareness of FP as a concept, understanding of the reasons for undergoing FP, knowledge of populations for whom FP is indicated, familiarity with specific FP methods, and understanding of the appropriate timing for seeking FP consultation.

### 2.3 Knowledge scoring systems

#### 2.3.1 College student knowledge scoring system (13-point scale)

A 13-point knowledge scoring system was constructed for the college student sample by integrating items from Sections B and C of the questionnaire. The scoring system comprised two domains. Domain 1 (basic reproductive health knowledge, 10 points) included: correct recognition that medical interventions cannot fully restore already diminished fertility (1 point); correct identification of the trend in infertility prevalence (1 point); and identification of factors contributing to fertility decline, scored cumulatively across seven items (cancer radiotherapy and chemotherapy, autoimmune disease, advanced age, environmental pollution, unhealthy diet, high-risk occupations, and reproductive system diseases; 1 point each, maximum 7 points); and self-reported understanding of the concept of fertility (1 point). Domain 2 (FP-specific knowledge, 3 points) included self-reported awareness of FP, understanding of the reasons for undergoing FP, and awareness of specific FP methods (1 point each). The total score ranged from 0 to 13, with higher scores indicating greater overall knowledge of reproductive health and FP. Construct validity was assessed by comparing knowledge scores between students who reported FP awareness and those who did not, with significantly higher scores observed in the FP-aware group.

#### 2.3.2 General population knowledge scoring system (11-point scale)

For analyses of members of the general population, an 11-point knowledge scoring system was developed using items that were common to both survey versions or could be harmonized across groups. Each item was scored as 1 point for a correct or affirmative response. The scale included: self-reported understanding of fertility; identification of six factors contributing to fertility decline (1 point each); understanding of the reasons for undergoing FP; knowledge of populations for whom FP is indicated; awareness of specific FP methods; and understanding of the appropriate timing for seeking FP consultation. Total scores ranged from 0 to 11, with higher scores indicating greater knowledge. Construct validity was again supported by significantly higher mean scores among participants who reported FP awareness compared with those who did not. Internal consistency was assessed using Cronbach’s *α*, yielding values of 0.708 for the 13-point college student scale and 0.783 for the 11-point general population scale, indicating acceptable reliability. Corrected item-total correlations ranged from 0.05 to 0.48 (college) and 0.33 to 0.52 (general). Because the scoring systems comprise factual knowledge items with objectively correct answers rather than attitudinal items reflecting a single latent construct, internal consistency is reported as a reference metric; construct validity (known-groups comparison) provides more appropriate evidence of measurement adequacy for knowledge assessments(see Supplementary Table S3 for item-level analysis).

### 2.4 Statistical analysis

#### 2.4.1 Descriptive analysis

All statistical analyses were performed using SPSS version 20.0 (IBM Corp., Armonk, NY, USA) and Python 3.12. A two-sided *p*-value < 0.05 was considered statistically significant. Descriptive statistics were used to characterize the study samples and summarize key questionnaire responses. Categorical variables were presented as frequencies and percentages. Continuous variables, including knowledge scores, were summarized as means with standard deviations and medians with ranges. The distribution of knowledge scores was examined in both the college student and general population samples. Independent-samples t-tests were used to compare mean knowledge scores between participants who reported FP awareness and those who did not.

#### 2.4.2 Bivariate analysis

Associations between FP awareness or willingness to use FP (the two primary outcome variables) and potential predictors were first examined using bivariate analyses. Chi-square tests were used for categorical variables, and independent-samples t-tests or Mann–Whitney U tests were used for continuous variables, as appropriate. Crude odds ratios (ORs) with 95% confidence intervals (CIs) were calculated for each potential predictor. Variables that showed statistically significant associations at *p* < 0.05, or were deemed clinically or epidemiologically important based on prior literature (e.g., gender, age, educational level), were considered candidates for inclusion in the multivariable models.

#### 2.4.3 Multivariable logistic regression

Multivariable logistic regression models were constructed to identify independent predictors of FP awareness and willingness to use FP, adjusting for potential confounders. Four separate models were fitted.

Model 1 (college students – FP awareness): The dependent variable was FP awareness (aware = 1, not aware = 0). Independent variables included gender, only-child status, place of residence (rural village, rural town, urban area), academic major (medical vs. non-medical), educational level (undergraduate vs. postgraduate), relationship status (single, in a relationship, married), history of premarital sexual activity, contraceptive use, and the 13-point knowledge score (continuous).

Model 2 (college students – FP willingness): The dependent variable was willingness to use FP services (willing = 1, not willing = 0). Independent variables included all covariates from Model 1 plus FP awareness status (aware vs. not aware).

Model 3 (general population – FP awareness): The dependent variable was FP awareness (aware = 1, not aware = 0). Independent variables included gender, age group (18–29, 30–39, 40–45, >45 years), educational attainment (ordinal), only-child status, medical professional background (yes vs. no), and the 11-point knowledge score (continuous).

Model 4 (general population – FP willingness): The dependent variable was willingness to use FP services (willing = 1, not willing = 0). Independent variables included gender, age group, educational attainment, marital status (married vs. unmarried), personal income, FP awareness status, and the 11-point knowledge score (continuous). In initial models, parental status (having children vs. no children) was also examined but was not statistically significant (adjusted OR = 1.42, *p* = 0.648) and had negligible impact on the estimated effects of other predictors. To maximize model parsimony and statistical efficiency, parental status was therefore excluded from the final Model 4.

For all models, adjusted odds ratios (aORs) with 95% CIs were reported. Model fit was assessed using the Hosmer-Lemeshow goodness-of-fit test with 10 groups and the area under the receiver operating characteristic curve (AUC). Multicollinearity among predictors was evaluated using variance inflation factors (VIF), with VIF > 5 considered indicative of problematic collinearity.

To address Objective 5, the patterns of independent predictors were compared descriptively across the college student sample (Models 1 and 2) and the general population sample (Models 3 and 4). Specifically, the direction, magnitude, and statistical significance of adjusted odds ratios for key predictors (knowledge score, medical background, gender, and other covariates) were compared across corresponding models to assess whether the determinants of FP awareness and willingness operated consistently across population groups. This comparison was conducted qualitatively rather than through formal statistical interaction testing, because the two sets of models were fitted to different samples using partially different covariate sets and knowledge scoring instruments (13-point scale for college students vs. 11-point scale for the general population), which precluded direct pooling of data or formal cross-model interaction terms. The rationale for this descriptive approach and its limitations are discussed in §4.8 and §4.11.

#### 2.4.4 Population attributable fraction (PAF) analysis

To quantify the population-level impact of modifiable predictors, population attributable fractions (PAFs) were calculated for variables that remained statistically significant in the multivariable models. The PAF was defined as the proportion of the outcome (e.g., lack of FP awareness or unwillingness to use FP) in the population that could theoretically be prevented if the exposure were eliminated or optimized. For categorical exposures, PAFs were estimated using the Levin formula:


$$\mathrm{PAF}=\frac{P_{e}\times (\text{OR}-1)}{P_{e}\times (\text{OR}-1)+1}$$

Where P_e is the prevalence of the exposure and OR is the adjusted odds ratio from the corresponding multivariable model. For protective factors (OR < 1), PAFs were interpreted as the proportion of the adverse outcome that could be reduced if the protective factor were universally adopted. For continuous predictors such as knowledge score, PAFs were estimated by comparing outcomes at different points in the score distribution (e.g., first vs. third quartile), using the corresponding OR per unit or per interquartile-range increase. Because FP willingness prevalence exceeded 50% in both samples, adjusted ORs may overestimate relative risks for these outcomes. As a sensitivity analysis, PAFs for FP willingness models (Models 2 and 4) were recalculated using corrected risk ratios derived from the Zhang and Yu formula: RR = OR / [(1 − P₀) + (P₀ × OR)], where P₀ is the outcome prevalence in the unexposed group (29). For FP awareness models (prevalence 21–26%), ORs approximate relative risks and correction was unnecessary. Results of the sensitivity analysis are presented in Supplementary Table S1.

#### 2.4.5 Handling of missing data

Missing data were handled using listwise deletion in both bivariate and multivariable analyses. The proportion of missing data was assessed for each variable prior to analysis. In the college student sample, 7 participants (0.92%) had missing data on key variables and were excluded, yielding an analytic sample of 758 students. In the general population sample, 6 participants were excluded because they were aged under 18 years and did not meet the inclusion criteria. An additional 1 participant had missing data on the FP awareness variable, resulting in an analytic sample of 1,001 for FP awareness (Model 3). For Model 4, 3 further participants had missing data on FP willingness or knowledge score items, resulting in an analytic sample of 998 for FP willingness (Model 4). Given the very low level of missingness (<1% for key variables), the impact of listwise deletion on estimates was expected to be minimal (Figure 1). As noted above, parental status was initially considered as a predictor in the FP willingness model but was excluded from the final model because it was non-significant and did not materially alter the effects of other covariates.

#### 2.4.6 Addressing potential sources of bias

Several strategies were employed to minimize potential sources of bias. Selection bias was mitigated through the use of stratified sampling across key demographic strata (residence type, gender, educational attainment, and academic major or occupational field), combined with a dual-mode data collection approach (online and paper-based) to reduce underrepresentation of individuals with limited internet access. Information bias was addressed through careful questionnaire development, including a review of existing instruments, adaptation to the Chinese sociocultural context, and pilot testing among 758 individuals to improve item clarity and reduce ambiguity. All knowledge items were based on established clinical evidence, and response options were designed to minimize acquiescence bias. Social desirability bias was mitigated by ensuring complete anonymity of responses; no personally identifiable information was collected, stored, or linked to survey data at any stage. Recall bias was expected to be minimal, as the questionnaire primarily assessed current knowledge and attitudes rather than past behaviors, with the exception of items on premarital sexual activity and contraceptive use, which may be subject to underreporting. Confounding was addressed through multivariable logistic regression, which adjusted simultaneously for all candidate predictors identified through bivariate analyses and prior literature review. Sensitivity analyses were conducted to assess the robustness of PAF estimates: for FP willingness models (outcome prevalence >50%), adjusted ORs were converted to corrected risk ratios using the Zhang and Yu formula before PAF recalculation (Supplementary Table S1). Additionally, the consistency of the principal findings across two independently sampled population groups (college students and the general population), analyzed using different but related knowledge scoring systems, provides indirect evidence of the robustness of the results.

### 2.5 Ethical considerations

The study protocol was reviewed and approved by the Reproductive Medicine Ethics Committee of Henan Provincial People’s Hospital (Approval Number: SYSZ-LL-2019012410). All participants received written or electronic information describing the study objectives, procedures, voluntary nature of participation, and data confidentiality measures before completing the questionnaire. Survey completion was entirely voluntary, and participants were free to withdraw at any time without consequence. No monetary or material incentives were offered for participation. All responses were collected anonymously, and no personally identifiable information was collected, stored, or linked to survey data at any stage of the study. Data were stored securely on password-protected servers with access restricted to authorized members of the research team.

## 3 Results

### 3.1 Demographic characteristics of the college student sample

A total of 765 college students completed the questionnaire. After excluding 7 participants with missing data on key variables, the effective analytic sample for the college student analyses (Models 1 and 2) comprised 758 students (Table 1). The demographic characteristics of this sample are summarized below.

**Table 1:** Demographic characteristics of college student participants in Henan Province (*n* = 758).

Characteristics	*n*	%
Gender		
Male	270	35.6
Female	488	64.4
Only Child		
Yes	87	11.5
No	671	88.5
Place of residence		
Urban	193	25.5
Rural town	190	25.1
Village	375	49.5
Academic major		
Science	196	25.9
Liberal arts	166	21.9
Medical-related	396	52.2
Education level		
Associate degree	23	3.0
Undergraduate	711	93.8
Postgraduate	24	3.2
Relationship status		
Single	544	71.7
In a relationship	210	27.7
Married	4	0.5
Premarital sexual activity		
Yes	97	12.8
No	661	87.2
Contraceptive use		
Yes	664	87.6
No	94	12.4

Data are presented as *n* (%). Analytic sample after exclusion of 7 participants with missing data on key variables from an initial sample of 765. Premarital sexual activity and contraceptive use are included as behavioral characteristics relevant to subsequent regression analyses.

The sample was predominantly female, with 488 women (64.4%) and 270 men (35.6%). The majority of participants (88.5%, *n* = 671) reported having at least one sibling, while 87 participants (11.5%) were only children, reflecting the family structure characteristic of this cohort in Henan Province. With respect to place of residence, 375 students (49.5%) originated from rural villages, 190 (25.1%) from rural towns, and 193 (25.5%) from urban areas, indicating that nearly half of the sample had a rural background. By academic discipline, medical students constituted the largest group (52.2%, *n* = 396), followed by science students (25.9%, *n* = 196) and liberal arts students (21.9%, *n* = 166), indicating that slightly more than half of the sample had exposure to medical education content. The vast majority of participants (93.8%, *n* = 711) were enrolled in undergraduate programs.

Regarding relationship status and sexual health behaviors, the majority of students were single (71.7%, *n* = 544), 27.7% (*n* = 210) reported being in a romantic relationship, and 0.5% (*n* = 4) were married. Approximately 12.8% (*n* = 97) reported a history of premarital sexual activity, and a substantial majority (87.6%, *n* = 664) reported actively using contraceptive methods.

### 3.2 Knowledge of fertility-related issues among college students

College students’ knowledge of fertility-related topics is summarized in Table 2, revealing both areas of adequate understanding and notable knowledge gaps.

**Table 2:** Fertility-related knowledge among college students in Henan Province (*n* = 758).

Knowledge item	Correct rate *n* (%)	Incorrect rate *n* (%)
Understanding of female physiological cycle and fertile period	484 (63.9%)	274 (36.1%)
Correct identification of optimal reproductive age for women (25–29 years)	560 (73.9%)	198 (26.1%)
Recognition of significant fertility decline at ages 35–39	267 (35.2%)	491 (64.8%)
Understanding that medical intervention cannot fully restore diminished fertility	438 (57.8%)	320 (42.2%)
Awareness that infertility rates are increasing	454 (59.9%)	304 (40.1%)

Correct responses based on current clinical evidence. Knowledge score (13-point scale) comprises 10 items on basic reproductive health knowledge and 3 items on FP-specific knowledge. FP-aware students scored significantly higher than non-aware students (10.31 ± 2.47 vs. 7.33 ± 2.07, *p* < 0.001).

A majority of respondents demonstrated basic understanding of key aspects of reproductive physiology. Specifically, 63.9% (*n* = 484) correctly identified the fertile period within the menstrual cycle, and 73.9% (*n* = 560) recognized the age range of 25 to 29 years as the optimal childbearing age for women. These findings suggest relatively good awareness of fundamental reproductive timing among this population.

However, substantial knowledge deficiencies were identified in several important domains. Only 35.2% (*n* = 267) of students correctly indicated that female fertility declines significantly between the ages of 35 and 39 years, while the remaining 64.8% (*n* = 491) selected incorrect age ranges, indicating widespread misconceptions about the onset and timing of clinically significant age-related fertility decline. When asked whether medical interventions can fully restore fertility that has already diminished, 57.8% (*n* = 438) correctly answered “no,” whereas 42.2% (*n* = 320) erroneously believed that medical technology could fully restore diminished fertility. Approximately 59.9% (*n* = 454) were aware that infertility rates have been increasing in recent years, indicating that a majority of students recognized broader population-level trends, even though their understanding of the underlying biological mechanisms remained incomplete.

**Figure 2:**
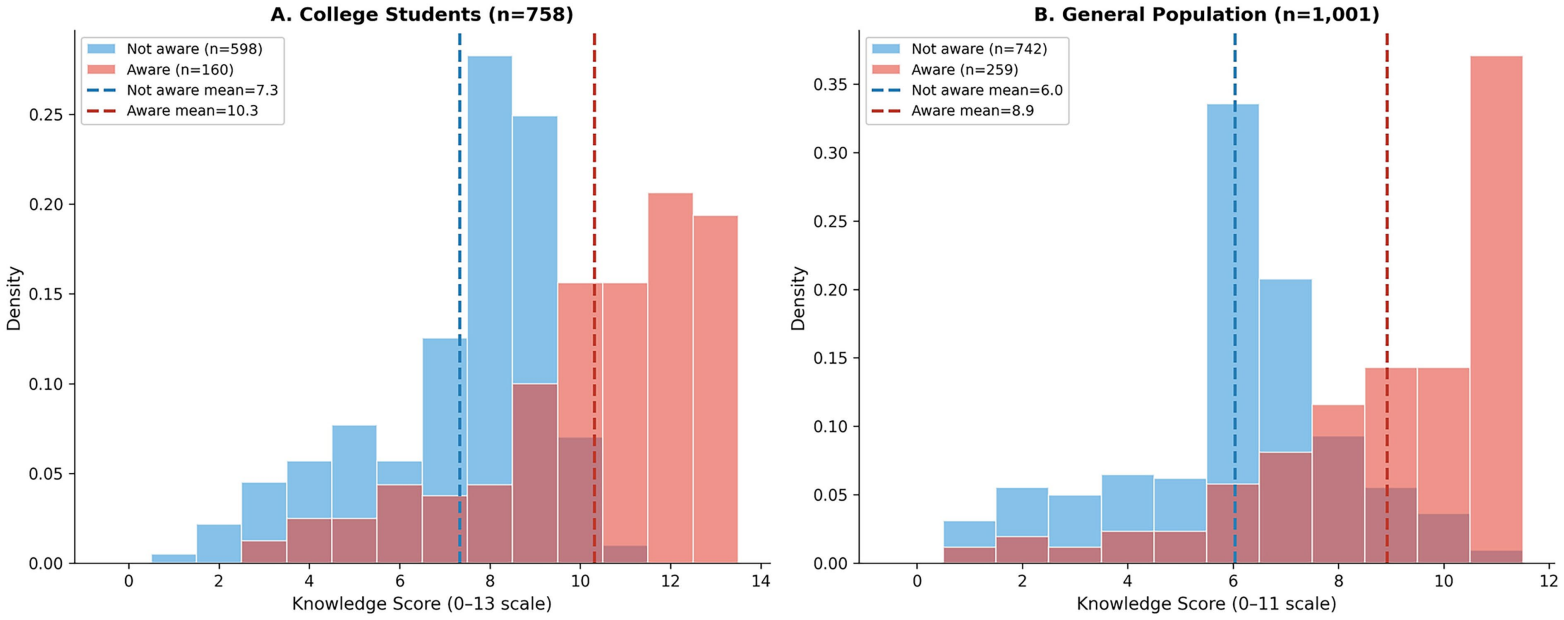
Distribution of knowledge scores by FP awareness status. **(A)** College students (*n* = 758): 13-point knowledge score distribution. Students aware of FP (red, *n* = 160) scored significantly higher than those not aware (blue, *n* = 598): mean 10.31 ± 2.47 vs. 7.33 ± 2.07 (*p* < 0.001). **(B)** General population (*n* = 1,001): 11-point knowledge score distribution. Participants aware of FP (red, *n* = 259) scored significantly higher than those not aware (blue, *n* = 742): mean 8.93 ± 2.38 vs. 6.04 ± 2.08 (*p* < 0.001). Dashed vertical lines indicate group means. The clear separation between distributions supports the construct validity of both scoring systems and the central role of knowledge in determining FP awareness.

The 13-point knowledge score among college students had a mean of 7.96 ± 2.48 (media*n* = 8, range = 1–13). Students who reported awareness of FP had significantly higher mean knowledge scores than those who did not (10.31 ± 2.47 vs. 7.33 ± 2.07; t = 15.49, *p* < 0.001), supporting the construct validity of the scoring system and suggesting that knowledge level is closely linked to FP awareness in this population (Figure 2).

Taken together, these findings indicate that college students in Henan Province possess relatively good knowledge of optimal reproductive timing but have limited understanding of the dynamics of age-related fertility decline and the realistic limitations of medical interventions for restoring diminished fertility.

### 3.3 Attitudes toward delayed childbearing and responses to potential infertility among college students

Attitudes toward potential infertility and delayed childbearing are summarized in Table 3 and provide contextual information for interpreting gender-related differences in FP willingness.

**Table 3:** Anticipated responses to potential infertility among college students by gender.

Variables	Males (*n* = 270)	Females (*n* = 488)
Assisted reproductive technology	108 (40.0)	150 (30.7)
Adoption	51 (18.9)	116 (23.8)
Choose childlessness	59 (21.9)	178 (36.5)
Continuous natural pregnancy attempts	52 (19.3)	44 (9.0)

Percentages calculated within each gender group. Respondents selected one option. Comparison between gender groups: χ^2^ test, *p* < 0.001.

Among male students (*n* = 270), the most commonly endorsed response was consideration of assisted reproductive technology (40.0%, *n* = 108), followed by acceptance of remaining childless (21.9%, *n* = 59), continuation of attempts at natural conception (19.3%, *n* = 52), and consideration of adoption (18.9%, *n* = 51). These distributions suggest that male students were somewhat more inclined to pursue technological solutions to infertility.

Among female students (*n* = 488), the most commonly endorsed response was acceptance of childlessness (36.5%, *n* = 178), followed by consideration of assisted reproductive technology (30.7%, *n* = 150), consideration of adoption (23.8%, *n* = 116), and continuation of attempts at natural conception (9.0%, *n* = 44). In notable contrast to their male counterparts, a substantially larger proportion of female students appeared prepared to accept the possibility of not having children.

Comparing the two groups, female students were markedly more likely than male students to endorse acceptance of childlessness (36.5% vs. 21.9%) and less likely to choose assisted reproductive technology as their primary response (30.7% vs. 40.0%). These gender-specific attitudinal patterns suggest that female students may be more inclined to adjust their life expectations and accept alternative life trajectories, whereas male students may place greater emphasis on actively pursuing medical solutions to maintain the possibility of biological parenthood.

### 3.4 Fertility preservation awareness among college students: bivariate analysis

**Figure 3:**
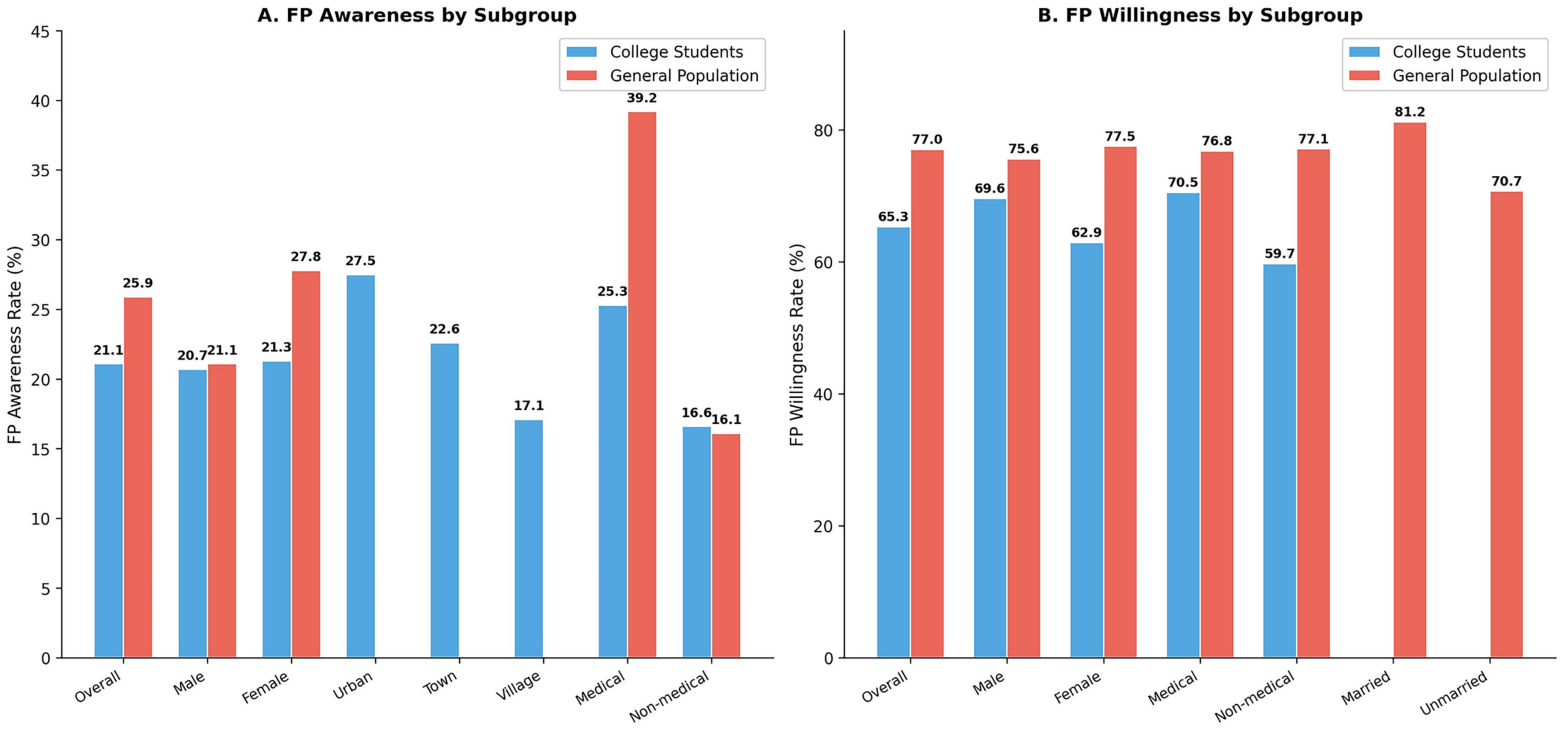
FP awareness and willingness rates by key demographic characteristics. **(A)** FP awareness rates by gender, place of residence (college students only), and medical/non-medical background. Awareness was generally low across all subgroups (< 30%), with the highest rates among participants with medical backgrounds. Residence data were not available for the general population sample in comparable form. **(B)** FP willingness rates by gender, medical background, and marital status (general population only). Willingness was higher overall than awareness, ranging from approximately 60 to 82% across subgroups. Blue bars represent college students; red bars represent the general population.

FP awareness among college students, examined by demographic characteristics using chi-square tests (Table 4 and Figure 3A), revealed several significant associations. Overall, 21.1% (*n* = 160) of college students reported having heard of FP, whereas 78.9% (*n* = 598) had not, indicating a generally low level of awareness in this population.

**Table 4:** Fertility preservation awareness among college students by demographic characteristics (*n* = 758).

Variables	Total *n*	Aware *n* (%)	Unaware *n* (%)	*p* value
Overall	758	160 (21.1)	598 (78.9)	
Gender				
Male	270	56 (20.7)	214 (79.4)	0.930
Female	488	104 (21.3)	384 (78.7)	
Only child status				
Yes	87	20 (23.0)	67 (77.0)	0.750
No	671	140 (20.9)	531 (79.1)	
Place of residence				
Urban	193	53 (27.5)	140 (72.5)	**0.010**
Rural town	190	43 (22.6)	147 (77.4)	
Village	375	64 (17.1)	311 (82.9)	
Academic major				
Science	196	31 (15.8)	165 (84.2)	**0.010**
Liberal arts	166	29 (17.5)	137 (82.5)	
Medical-related	396	100 (25.3)	296 (74.8)	
Education level				
Associate	23	6 (26.1)	17 (73.9)	0.740
Undergraduate	711	148 (20.8)	563 (79.2)	
Postgraduate	24	6 (25.00)	18 (75.00)	

*p*-values derived from chi-square tests. F*P* = fertility preservation. All 758 participants had complete data on the FP awareness variable; no additional exclusions were necessary for this analysis.

Bold values indicate *p* < 0.05.

FP awareness varied significantly by place of residence (*p* = 0.013). Urban students had the highest awareness rate (27.5%, *n* = 53), followed by students from rural towns (22.6%, *n* = 43) and students from rural villages (17.1%, *n* = 64), suggesting that contextual factors related to the living environment, such as access to health information and proximity to specialized medical services, may influence exposure to FP-related knowledge.

Academic major was also significantly associated with FP awareness (*p* = 0.013). Among medical students, 25.3% (*n* = 100) reported having heard of FP, compared with 17.5% (*n* = 29) of liberal arts students and 15.8% (*n* = 31) of science students. This pattern is consistent with the expectation that medical curricula provide greater exposure to reproductive health topics, although the absolute level of awareness even among medical students remained relatively low.

No statistically significant differences in FP awareness were observed by gender (*p* = 0.93), only-child status (*p* = 0.75), or educational level (*p* = 0.74) in the bivariate analysis, suggesting that these demographic factors did not play a major independent role in shaping FP awareness among college students at the bivariate level. These bivariate associations motivated inclusion of residence, academic major, sexual health behaviors, and knowledge score as candidate predictors in the multivariable model for FP awareness.

### 3.5 Demographic characteristics of the general population sample

The general population sample included 1008 participants (Table 5), providing a broad cross-section of adults residing in Henan Province. After exclusion of 6 participants aged under 18 years and a small number with missing data on key variables, 1,001 respondents were included in the FP awareness analysis (Model 3) and 998 in the FP willingness analysis (Model 4). Over half of the sample (51.1%, *n* = 515) were aged 18 to 29 years, 40.3% (*n* = 406) were aged 30 to 39 years, and 8.1% (*n* = 81) were aged 40 years or older. Women constituted 69.8% (*n* = 704) of respondents, which may reflect greater willingness among women to participate in surveys on reproductive health topics.

**Table 5:** Demographic characteristics of general population participants in Henan Province (*N* = 1,008).

Characteristics	*n*	%
Age		
<18 years	6	0.6
18–29 years	515	51.1
30–39 years	406	40.3
40–45 years	59	5.9
>45 years	22	2.2
Gender		
Male	303	30.1
Female	704	69.8
Education level		
High School or below	180	17.9
Undergraduate	476	47.2
Postgraduate	352	34.9
Only child status		
Yes	194	19.2
No	811	80.5
Marital status		
Married	597	59.2
Unmarried	409	40.6

Data are presented as n (%). Totals for individual variables may not sum to *N* = 1,008 due to missing data on selected items (1 participant missing gender data, 3 missing only-child status, 2 missing marital status, 6 missing age data). Six participants aged < 18 years were excluded from analytic models (Models 3 and 4) per inclusion criteria. See Figure 1 for participant flow.

Educational attainment in the sample was relatively high: 47.2% (*n* = 476) held an undergraduate degree, 34.9% (*n* = 352) had completed postgraduate education, and 17.9% (*n* = 180) had completed education up to the high school level. The majority of participants (80.5%, *n* = 811) reported having at least one sibling, and 59.2% (*n* = 597) were married, providing important contextual information for interpreting their fertility-related attitudes and willingness to consider FP services.

The proportion of women in our sample (69.8%) exceeds the Henan Province census proportion (approximately 49%), likely reflecting greater willingness among women to participate in surveys on reproductive health topics. The age distribution and educational attainment of the sample are broadly consistent with the demographic profile of urban and peri-urban populations in Henan, though rural populations may be underrepresented.

### 3.6 Willingness to use fertility preservation services in the general population: bivariate analysis

Willingness to use FP services among members of the general population, stratified by key demographic characteristics (Table 6 and Figure 3B), is summarized below. Responses were categorized as “willing,” “unwilling,” or “uncertain”; in subsequent regression analyses (Model 4, Table 10), “unwilling” and “uncertain” responses were combined as “not willing.”

**Table 6:** Attitudes toward fertility preservation among general population by demographic characteristics (*N* = 1,008).

Characteristics	*n*	Willing	Unwilling	Uncertain	X^2^	*p*-value
Gender						
Male	302	228(75.5)	21(7.0)	53(17.5)	0.95	0.621
Female	702	545(77.6)	51(7.3)	106(15.1)		
Education level						
High School or below	180	134(74.4)	6(3.3)	40(22.2)	14.17	**0.007**
Undergraduate	475	368(77.5)	32(6.7)	75(15.8)		
Postgraduate	350	272(77.7)	34(9.7)	44(12.6)		
Monthly personal income						
<2000 RMB	267	187(70.0)	32(12.0)	48(18.0)	9.39	0.052
2000–8,000 RMB	269	205(76.2)	17(6.3)	47(17.5)		
>8,000 RMB	191	153(80.1)	13(6.8)	25(13.1)		
Parental status						
Has children	324	257(79.3)	16(4.9)	51(15.7)	1.09	0.580
No children	278	229(82.4)	10(3.6)	39(14.0)		

Data are presented as *n* (row %). Willingness was defined as responding ‘willing’ to Q27; ‘Unwilling’ and ‘Uncertain’ responses were combined as ‘not willing’ in regression analyses (Models 4, Table 10). Chi-square tests compare the distribution of willing/unwilling/uncertain across categories. 3 participants excluded for missing willingness data.

Bold values indicate *p* < 0.05.

Overall, approximately 77.0% of respondents with available data expressed willingness to use FP services, 7.2% indicated unwillingness, and 15.8% reported uncertainty. This overall level of willingness was considerably higher than the rate of FP awareness (25.9%), suggesting that many individuals who had not previously heard of FP were nonetheless open to considering it once the concept was introduced.

Gender was not significantly associated with the distribution of FP willingness responses (χ^2^ = 0.95, *p* = 0.621): willingness rates were similar between men (75.5%, *n* = 228/302) and women (77.6%, *n* = 545/702). This finding contrasts with patterns observed in the college student sample and in prior literature, and may reflect the influence of other sociodemographic factors that differ across groups.

Educational attainment was significantly associated with FP-related attitudes (χ^2^ = 14.17, *p* = 0.007). Willingness rates were similar across educational levels-74.4% among those with a high school education or below, 77.5% among those with an undergraduate degree, and 77.7% among those with postgraduate education-but the proportion reporting explicit unwillingness increased with educational level (3.3, 6.7, and 9.7%, respectively), while the proportion reporting uncertainty decreased correspondingly (22.2, 15.8, and 12.6%). This pattern suggests that higher education may be associated with more definitive attitudes-whether positive or negative-rather than uniformly increasing willingness.

Monthly personal income showed a positive gradient with willingness: 70.0% of participants earning less than 2,000 RMB per month expressed willingness, compared with 76.2% of those earning 2,000–8,000 RMB and 80.1% of those earning more than 8,000 RMB, suggesting that greater financial resources may lower perceived barriers to FP utilization. Among participants with available parental status data, willingness rates were comparable between those with children (79.3%, *n* = 257/324) and those without children (82.4%, *n* = 229/278).

These bivariate findings indicate that willingness to use FP services in the general population is relatively high overall and varies by educational attainment and income level, but does not differ significantly by gender. However, because these factors are likely to be intercorrelated, multivariable analysis was required to identify which factors operate as independent predictors (see §3.10). Although parental status showed comparable willingness rates across categories in bivariate analyses, its effect did not remain significant after adjustment for other variables in the multivariable model (aOR = 1.42, *p* = 0.648) and was therefore not retained in the final Model 4.

### 3.7 Multivariable logistic regression and PAF analysis: FP awareness among college students

**Figure 4:**
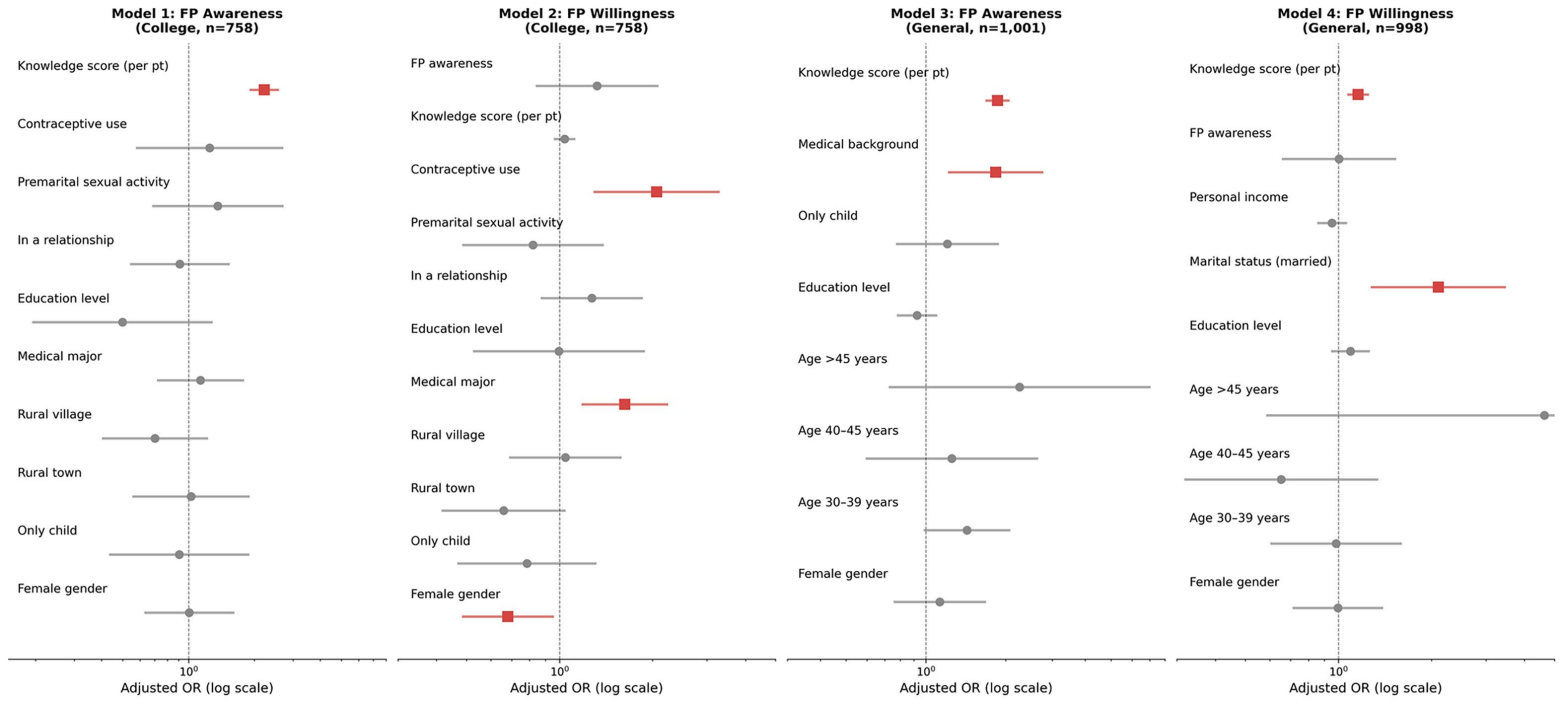
Forest plot of adjusted odds ratios from multivariable logistic regression models. Adjusted odds ratios (aORs) with 95% confidence intervals are shown for each predictor variable across four models. Model 1: FP awareness among college students (*n* = 758). Model 2: FP willingness among college students (*n* = 758). Model 3: FP awareness in the general population (*n* = 1,001). Model 4: FP willingness in the general population (*n* = 998). Red squares indicate statistically significant predictors (*p* < 0.05); gray circles indicate non-significant predictors. The vertical dashed line represents OR = 1.0 (null effect). The married variable (*n* = 4) was excluded from the Model 1 panel due to estimation instability from quasi-complete separation. The *x*-axis is displayed on a logarithmic scale.

The results of the multivariable logistic regression analysis for FP awareness among college students (Model 1), together with corresponding PAF estimates, are presented in Table 7 and Figure 4.

**Table 7:** Multivariable logistic regression and PAF analysis for FP awareness among college students (*n* = 758).

Variable	Category	*n* (%)	Crude OR	95% CI	*p* value	Adjusted OR	95% CI	*p* value	PAF (%)	PAF *p*
Gender	Male	270 (35.6)	1.00 (ref)	---	---	1.00 (ref)	---	---	---	---
	Female	488 (64.4)	1.03	0.72–1.49	0.854	1.01	0.63–1.60	0.976	---	---
Only child	No	671 (88.5)	1.00 (ref)	---	---	1.00 (ref)	---	---	---	---
	Yes	87 (11.5)	1.13	0.66–1.93	0.648	0.91	0.44–1.87	0.789	---	---
Place of residence	Urban	193 (25.5)	1.00 (ref)	---	---	1.00 (ref)	---	---	---	---
	Rural town	190 (25.1)	1.13	0.76–1.68	0.552	1.03	0.56–1.88	0.935	---	---
	Rural village	375 (49.5)	0.62	0.43–0.88	**0.007**	0.70	0.41–1.21	0.203	---	---
Academic major	Non-medical	362 (47.8)	1.00 (ref)	---	---	1.00 (ref)	---	---	---	---
	Medical	396 (52.2)	1.70	1.19–2.43	**0.004**	1.13	0.72–1.77	0.586	---	---
Education level	---	---	0.97	0.48–1.96	0.940	0.50	0.19–1.27	0.145	---	---
Relationship status	Single	544 (71.7)	1.00 (ref)	---	---	1.00 (ref)	---	---	---	---
	In a relationship	210 (27.7)	1.24	0.85–1.82	0.260	0.91	0.55–1.52	0.721	---	---
	Married	4 (0.5)	3.77	0.53–26.99	0.186	42.95	2.13–864.58	**0.014**	18.1	**0.014**
Premarital sexual activity	No	661 (87.2)	1.00 (ref)	---	---	1.00 (ref)	---	---	---	---
	Yes	97 (12.8)	2.29	1.45–3.63	**<0.001**	1.36	0.69–2.68	0.374	---	---
Contraceptive use	No	94 (12.4)	1.00 (ref)	---	---	1.00 (ref)	---	---	---	---
	Yes	664 (87.6)	1.96	1.04–3.69	**0.037**	1.25	0.58–2.68	0.570	---	---
Knowledge score (0–13)	Per 1-point increase	---	2.22	1.94–2.55	**<0.001**	2.22	1.92–2.56	**<0.001**	62.8	**<0.001**

Outcome: FP awareness (aware = 1, not aware = 0). Awareness rate: 21.1% (160/758). OR = odds ratio; CI = confidence interval; PAF = population attributable fraction; ref = reference category. Knowledge score measured on a 13-point scale (0–13). PAF for knowledge score was calculated by comparing the first quartile (Q1 = 7) to the third quartile (Q3 = 9) of the score distribution using the Levin formula. Bold values indicate *p* < 0.05. The married category (*n* = 4) produced unstable estimates due to quasi-complete separation and should be interpreted with caution. Model diagnostics: Hosmer-Lemeshow χ^2^ = 162.21 (*p* < 0.001; note: H-L test is overpowered with large samples and strong continuous predictors), AUC = 0.844, all VIF < 1.72.

In the unadjusted (crude) analysis, several variables demonstrated statistically significant associations with FP awareness. Students from rural villages had lower odds of FP awareness compared with urban students (crude OR = 0.62, 95% CI: 0.43–0.88, *p* = 0.007). Medical students had higher odds of awareness than non-medical students (crude OR = 1.70, 95% CI: 1.19–2.43, *p* = 0.004). History of premarital sexual activity (crude OR = 2.29, 95% CI: 1.45–3.63, *p* < 0.001) and active contraceptive use (crude OR = 1.96, 95% CI: 1.04–3.69, *p* = 0.037) were also significantly associated with higher FP awareness. Knowledge score was strongly associated with awareness in the crude analysis (crude OR = 2.22, 95% CI: 1.94–2.55, *p* < 0.001).

However, in the multivariable model, which simultaneously adjusted for all candidate predictors, only one variable remained statistically significant: knowledge score (aOR = 2.22, 95% CI: 1.92–2.56, *p* < 0.001). This indicates that for each 1-point increase in the 13-point knowledge score, the odds of being aware of FP more than doubled. To contextualize this effect, an increase in knowledge score from the 25th percentile (7 points) to the 75th percentile (9 points) corresponded to an approximate five-fold increase in the odds of FP awareness (OR = 2.22^2^ ≈ 4.93), illustrating the magnitude of the knowledge gradient.

Notably, all other variables that were significant in the unadjusted analysis-including place of residence (rural village: aOR = 0.70, 95% CI: 0.41–1.21, *p* = 0.203), academic major (medical: aOR = 1.13, 95% CI: 0.72–1.77, *p* = 0.586), premarital sexual activity (aOR = 1.36, 95% CI: 0.69–2.68, *p* = 0.374), and contraceptive use (aOR = 1.25, 95% CI: 0.58–2.68, *p* = 0.570)-lost statistical significance after adjustment for knowledge score. This pattern is consistent with a mediating role of knowledge: the effects of rural residence, non-medical major, and sexual health behaviors on FP awareness appear to operate largely through their association with overall reproductive health and FP knowledge, rather than exerting independent effects.

Gender (aOR = 1.01, *p* = 0.976), only-child status (aOR = 0.91, *p* = 0.789), educational level (aOR = 0.50, *p* = 0.145), and relationship status (in a relationship: aOR = 0.91, *p* = 0.721) were not significantly associated with FP awareness in either the crude or adjusted analyses. The married category (*n* = 4) produced unstable estimates due to the very small cell size and should be interpreted with caution.

The PAF analysis further quantified the population-level impact of knowledge on FP awareness. The estimated PAF for knowledge score (comparing the first quartile to the third quartile of the score distribution, Q1 = 7, Q3 = 9) was 62.8% (*p* < 0.001), indicating that if all students’ knowledge levels were elevated from the lower range to the upper range, approximately 62.8% of the current lack of FP awareness could theoretically be prevented. No other variable yielded a statistically significant PAF, reinforcing the conclusion that knowledge is the single most important modifiable determinant of FP awareness in this population.

Taken together, these findings indicate that knowledge is the dominant modifiable determinant of FP awareness in the college student population, whereas other demographic and behavioral variables exert little independent influence after accounting for knowledge level.

### 3.8 Multivariable logistic regression and PAF analysis: FP willingness among college students

The multivariable logistic regression analysis for willingness to use FP services among college students (Model 2), along with PAF estimates, is presented in Table 8.

**Table 8:** Multivariable logistic regression and PAF analysis for FP willingness among college students (*n* = 758).

Variable	Category	*n* (%)	Crude OR	95% CI	*p* value	Adjusted OR	95% CI	*p* value	PAF (%)	PAF *p*
Gender	Male	270 (35.6)	1.00 (ref)	---	---	1.00 (ref)	---	---	---	---
	Female	488 (64.4)	0.74	0.54–1.02	0.063	0.68	0.49–0.95	**0.024**	---	**0.024**
Only child	No	671 (88.5)	1.00 (ref)	---	---	1.00 (ref)	---	---	---	---
	Yes	87 (11.5)	0.90	0.57–1.44	0.664	0.78	0.47–1.30	0.348	---	---
Place of residence	Urban	193 (25.5)	1.00 (ref)	---	---	1.00 (ref)	---	---	---	---
	Rural town	190 (25.1)	0.67	0.48–0.94	**0.022**	0.66	0.42–1.04	0.071	---	---
	Rural village	375 (49.5)	1.18	0.87–1.59	0.278	1.04	0.69–1.57	0.837	---	---
Academic major	Non-medical	362 (47.8)	1.00 (ref)	---	---	1.00 (ref)	---	---	---	---
	Medical	396 (52.2)	1.61	1.19–2.18	**0.002**	1.62	1.19–2.22	**0.002**	24.6	**0.002**
Education level	---	---	0.94	0.52–1.71	0.841	0.99	0.53–1.87	0.987	---	---
Relationship status	Single	544 (71.7)	1.00 (ref)	---	---	1.00 (ref)	---	---	---	---
	In a relationship	210 (27.7)	1.30	0.92–1.83	0.131	1.27	0.88–1.84	0.205	---	---
	Married	4 (0.5)	1.60	0.17–15.43	0.686	3.33	0.29–38.76	0.336	---	---
Premarital sexual activity	No	661 (87.2)	1.00 (ref)	---	---	1.00 (ref)	---	---	---	---
	Yes	97 (12.8)	1.22	0.77–1.92	0.404	0.82	0.49–1.38	0.455	---	---
Contraceptive use	No	94 (12.4)	1.00 (ref)	---	---	1.00 (ref)	---	---	---	---
	Yes	664 (87.6)	1.97	1.28–3.05	**0.002**	2.06	1.30–3.27	**0.002**	48.2	**0.002**
Knowledge score (0–13)	Per 1-point increase	---	1.08	1.02–1.15	0.009	1.04	0.97–1.12	0.308	---	---
FP awareness	Not aware	598 (78.9)	1.00 (ref)	---	---	1.00 (ref)	---	---	---	---
	Aware	160 (21.1)	1.58	1.08–2.33	**0.020**	1.32	0.84–2.07	0.223	---	---

Outcome: FP willingness (willing = 1, not willing = 0). Willingness rate: 65.3% (495/758). OR = odds ratio; CI = confidence interval; PAF = population attributable fraction; ref = reference category. Bold values indicate *p* < 0.05. PAF calculated using the Levin formula: PAF = Pe × (OR − 1)/[Pe × (OR − 1) + 1], where Pe is exposure prevalence. Model diagnostics: Hosmer-Lemeshow χ^2^ = 7.09 (*p* = 0.527), AUC = 0.635, all VIF < 1.72.

In the unadjusted analysis, several variables were significantly associated with FP willingness. Medical major (crude OR = 1.61, 95% CI: 1.19–2.18, *p* = 0.002), active contraceptive use (crude OR = 1.97, 95% CI: 1.28–3.05, *p* = 0.002), FP awareness (crude OR = 1.58, 95% CI: 1.08–2.33, *p* = 0.020), and knowledge score (crude OR = 1.08, 95% CI: 1.02–1.15, *p* = 0.009) were all positively associated with willingness. Female gender showed a trend toward lower willingness that did not reach statistical significance in the crude analysis (crude OR = 0.74, 95% CI: 0.54–1.02, *p* = 0.063). Students from rural towns showed lower willingness compared with urban students (crude OR = 0.67, 95% CI: 0.48–0.94, *p* = 0.022).

In the multivariable model, three variables emerged as statistically significant independent predictors of FP willingness:

First, gender was independently associated with FP willingness, with female students showing significantly lower odds of willingness than male students (aOR = 0.68, 95% CI: 0.49–0.95, *p* = 0.024). This finding indicates that male college students were approximately 1.47 times more likely than female students to express willingness to use FP services, a finding that contrasts with the common assumption that women, as the primary bearers of age-related fertility decline, would be more receptive to FP. Notably, the gender effect became statistically significant only after adjustment for other covariates, suggesting that confounding by factors such as medical major and contraceptive use masked the gender difference in the crude analysis.

Second, medical major was a strong independent predictor (aOR = 1.62, 95% CI: 1.19–2.22, *p* = 0.003), indicating that students enrolled in medical programs had 62% higher odds of expressing willingness to use FP compared with students in non-medical disciplines. Unlike the finding for FP awareness, where the effect of medical major was fully mediated by knowledge score, the effect of medical major on FP willingness remained significant after controlling for knowledge, suggesting that medical education may influence willingness through pathways beyond factual knowledge, such as exposure to clinical settings, normalization of reproductive technologies, and greater comfort with medical procedures.

Third, active contraceptive use was a significant independent predictor (aOR = 2.06, 95% CI: 1.30–3.27, *p* = 0.002), indicating that students who reported actively using contraceptive methods had more than twice the odds of expressing willingness to use FP services compared with those who did not use contraception. This novel finding suggests a meaningful link between proactive reproductive health behavior (contraceptive use) and receptiveness to FP, potentially mediated by a broader orientation toward reproductive planning and health-conscious decision-making.

Notably, FP awareness (aOR = 1.32, 95% CI: 0.84–2.07, *p* = 0.223) and knowledge score (aOR = 1.04, 95% CI: 0.97–1.12, *p* = 0.309) were not statistically significant in the multivariable model for FP willingness. This contrasts with the findings for FP awareness (Section 3.7), where knowledge score was the sole independent predictor. The loss of significance for these variables in the willingness model suggests that their effects were explained by other factors in the model, particularly medical major and contraceptive use, or that the pathway from knowledge to willingness is more complex and may require additional mediating variables.

Residence in a rural town showed a borderline but non-significant association with lower willingness (aOR = 0.66, 95% CI: 0.42–1.04, *p* = 0.071), suggesting a potential urban–rural gradient in willingness that may warrant further investigation in larger studies. Only-child status (aOR = 0.78, *p* = 0.348), educational level (aOR = 0.99, *p* = 0.987), relationship status (aOR = 1.27, *p* = 0.205 for in a relationship), and premarital sexual activity (aOR = 0.82, *p* = 0.455) were not significantly associated with FP willingness in the adjusted analysis.

The PAF analysis identified two key modifiable factors with significant population-level impact on FP willingness. The largest PAF was observed for contraceptive use (PAF = 48.2%, *p* = 0.002): given the high prevalence of contraceptive use in this sample (87.6%), this finding indicates that proactive reproductive health behavior is associated with a substantial proportion of the population-level willingness to consider FP. Medical major also demonstrated a significant PAF of 24.6% (*p* = 0.002), indicating that the knowledge and attitudes acquired through medical education contribute meaningfully to FP willingness at the population level, and that extending similar educational content to non-medical students could substantially increase overall willingness.

### 3.9 Multivariable logistic regression: FP awareness in the general population

The general population analysis of FP awareness (Model 3; *n* = 1,001; Table 9) examined independent predictors using multivariable logistic regression.

**Table 9:** Multivariable logistic regression for FP awareness in the general population (*n* = 1,001).

Variable	Category	Crude OR	95% CI	*p* value	Adjusted OR	95% CI	*p* value
Gender	Male	1.00 (ref)	---	---	1.00 (ref)	---	---
	Female	1.42	1.03–1.96	**0.032**	1.13	0.76–1.67	0.545
Age group	18–29 years	1.00 (ref)	---	---	1.00 (ref)	---	---
	30–39 years	1.29	0.97–1.72	0.079	1.43	0.99–2.06	0.055
	40–45 years	1.39	0.79–2.45	0.254	1.25	0.60–2.62	0.551
	>45 years	2.02	0.85–4.78	0.110	2.26	0.73–6.97	0.157
Education level	Per level increase	1.31	1.17–1.48	**<0.001**	0.93	0.78–1.09	0.363
Only child	No	1.00 (ref)	---	---	1.00 (ref)	---	---
	Yes	1.25	0.88–1.78	0.209	1.21	0.78–1.87	0.401
Professional background	Non-medical	1.00 (ref)	---	---	1.00 (ref)	---	---
	Medical	3.35	2.49–4.50	**<0.001**	1.83	1.22–2.75	**0.003**
Knowledge score (0–11)	Per 1-point increase	1.93	1.76–2.11	**<0.001**	1.86	1.70–2.05	**<0.001**

**Figure 5:**
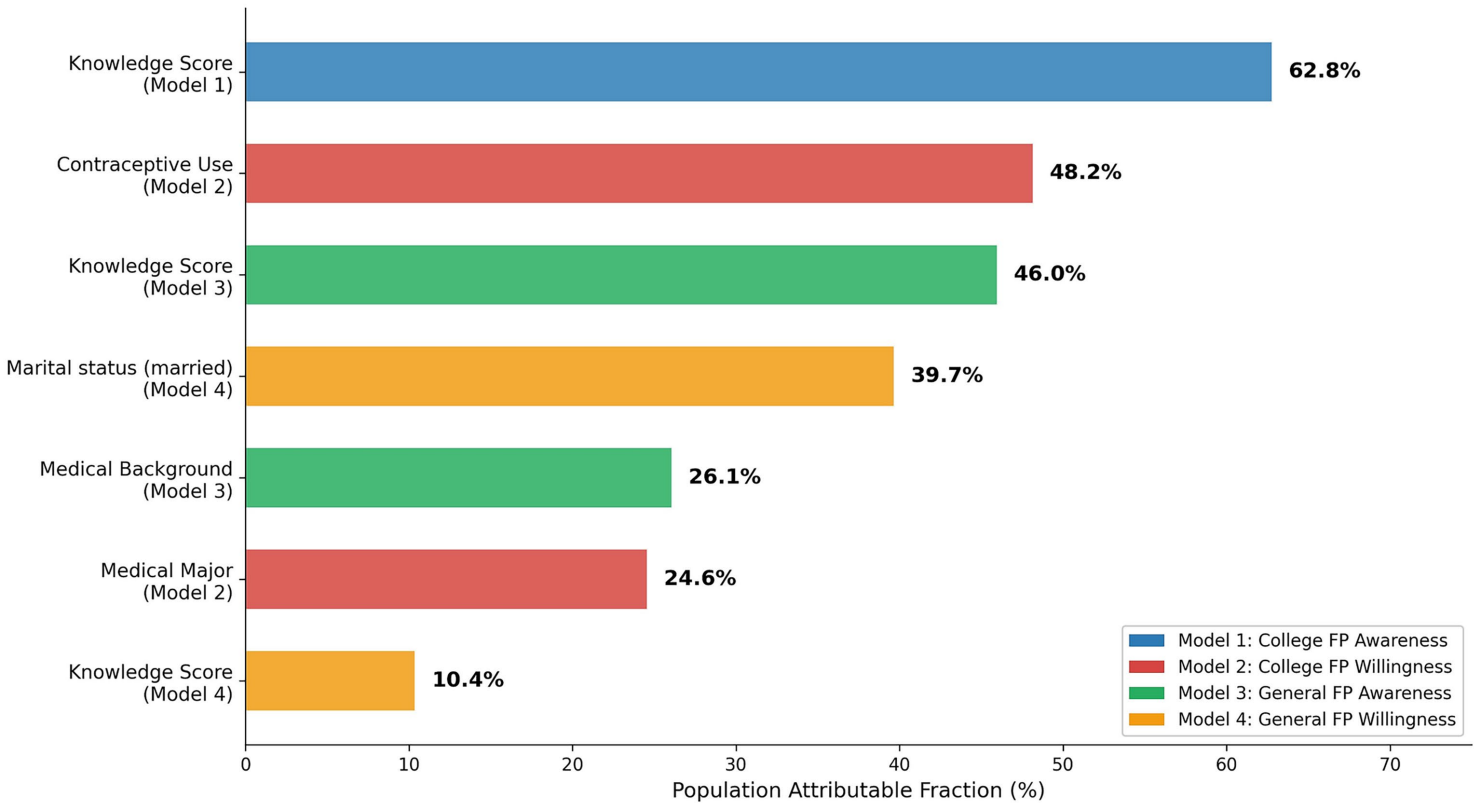
Population attributable fractions (PAF) of significant modifiable predictors. PAF estimates represent the proportion of the outcome (lack of FP awareness or unwillingness to use FP) that could theoretically be prevented if the modifiable factor were optimized across the population. PAFs were calculated using the Levin formula with adjusted odds ratios from the corresponding multivariable models. For knowledge score, PAF was estimated by comparing outcomes at the first quartile (Q1) versus the third quartile (Q3) of the score distribution. Colors indicate the source model. Knowledge score demonstrated the largest PAF for FP awareness (62.8%), while contraceptive use showed the largest PAF for FP willingness among college students (48.2%).

Outcome: FP awareness (aware = 1, not aware = 0). Awareness rate: 25.9% (259/1,001). Sample restricted to participants aged ≥ 18 years with non-missing FP awareness data. OR = odds ratio; CI = confidence interval; ref = reference category. Knowledge score measured on an 11-point scale (0–11). Bold values indicate *p* < 0.05. PAF estimates for significant predictors (knowledge score and medical background) are reported in the text (§3.9) and Figure 5. Model diagnostics: Hosmer-Lemeshow χ^2^ = 96.00 (*p* < 0.001; see text for interpretation), AUC = 0.838, all VIF < 1.49.

Overall, 25.9% (*n* = 259) of participants in the general sample reported awareness of FP, while 74.1% (*n* = 742) did not. The 11-point knowledge scoring system developed for the general population sample demonstrated strong construct validity, with FP-aware participants scoring significantly higher than non-aware participants (8.93 ± 2.38 vs. 6.04 ± 2.08; *p* < 0.001).

In the unadjusted analysis, several variables were significantly associated with FP awareness. Female gender (crude OR = 1.42, 95% CI: 1.03–1.96, *p* = 0.032), higher educational attainment (crude OR = 1.31, 95% CI: 1.17–1.48, *p* < 0.001), medical professional background (crude OR = 3.35, 95% CI: 2.49–4.50, *p* < 0.001), and higher knowledge score (crude OR = 1.93, 95% CI: 1.76–2.11, *p* < 0.001) were all positively associated with awareness. Age 30–39 years versus 18–29 years showed a borderline association (crude OR = 1.29, 95% CI: 0.97–1.72, *p* = 0.079) that did not reach statistical significance.

In the multivariable model, two variables emerged as statistically significant independent predictors of FP awareness in the general population:

First, medical professional background was independently associated with higher FP awareness (aOR = 1.83, 95% CI: 1.22–2.75, *p* = 0.003), indicating that individuals with a medical education or professional background had 83% higher odds of being aware of FP compared with those without a medical background, after adjusting for all other covariates including knowledge score. This suggests that medical training confers an awareness advantage that extends beyond the factual knowledge captured by the scoring system, potentially through exposure to clinical discussions, professional networks, or continuing medical education.

Second, knowledge score was the strongest independent predictor (aOR = 1.86, 95% CI: 1.70–2.05, *p* < 0.001), indicating that each 1-point increase on the 11-point knowledge scale was associated with an 86% increase in the odds of FP awareness. This finding is consistent with and corroborates the result observed in the college student-only analysis (aOR = 2.22 on the 13-point scale) (Table 7), confirming the central role of knowledge in driving FP awareness across different population groups and measurement instruments.

All other variables lost statistical significance in the multivariable model, including gender (aOR = 1.13, 95% CI: 0.76–1.67, *p* = 0.545), age group (aOR = 1.43, *p* = 0.055 for 30–39 years; aOR = 1.25, *p* = 0.551 for 40–45 years; aOR = 2.26, *p* = 0.157 for >45 years), educational attainment (aOR = 0.93, *p* = 0.363), and only-child status (aOR = 1.21, *p* = 0.401). The attenuation of the education effect after adjustment for knowledge score suggests that the influence of formal educational attainment on FP awareness is substantially mediated through the acquisition of reproductive health and FP-specific knowledge.

To quantify the population-level impact of the significant predictors identified above, PAF estimates were calculated using the adjusted odds ratios from Model 3 (Figure 5). Knowledge score was the modifiable factor with the greatest population-level impact: comparing outcomes at the first quartile versus the third quartile of the knowledge score distribution, the estimated PAF was approximately 46% (*p* < 0.001), indicating that elevating knowledge levels across the population could prevent a substantial proportion of the current lack of FP awareness. The PAF for medical background was also significant (*p* = 0.003), but its population-level impact was limited by the lower prevalence of medical training in the general population (42.3%).

The pattern of independent predictors observed in this general population model was broadly consistent with the findings from the college student-only analysis (Model 1, Table 7): in both analyses, knowledge score was the dominant predictor of FP awareness, and demographic variables such as gender, age, and educational attainment did not retain significance after adjustment. Medical background retained an independent effect in the general population model, consistent with the direction of the crude association observed among college students.

### 3.10 Multivariable logistic regression and PAF analysis: FP willingness in the general population

The general population analysis of willingness to use FP services (Model 4; *n* = 998; Table 10) identified marital status and knowledge score as independent predictors.

**Table 10:** Multivariable logistic regression and PAF analysis for FP willingness in the general population (*n* = 998).

Variable	Category	Crude OR	95% CI	*p* value	Adjusted OR	95% CI	*p* value	PAF (%)	PAF *p*
Gender	Male	1.00 (ref)	---	---	1.00 (ref)	---	---	---	---
	Female	1.12	0.81–1.53	0.502	0.99	0.72–1.38	0.976	---	---
Age group	18–29 years	1.00 (ref)	---	---	1.00 (ref)	---	---	---	---
	30–39 years	1.49	1.09–2.03	**0.012**	0.98	0.61–1.59	0.941	---	---
	40–45 years	0.79	0.44–1.44	0.445	0.65	0.32–1.33	0.240	---	---
	>45 years	6.44	0.86–48.12	0.070	4.63	0.59–36.49	0.145	---	---
Education level	Per level increase	1.05	0.94–1.16	0.409	1.09	0.95–1.25	0.198	---	---
Marital status	Unmarried	1.00 (ref)	---	---	1.00 (ref)	---	---	---	---
	Married	1.79	1.33–2.40	**<0.001**	2.10	1.28–3.44	**0.003**	39.7	**0.003**
Personal income	Per level increase	1.02	0.93–1.11	0.733	0.95	0.86–1.06	0.354	---	---
FP awareness	Not aware	1.00 (ref)	---	---	1.00 (ref)	---	---	---	---
	Aware	1.58	1.10–2.27	**0.014**	1.00	0.66–1.52	0.984	---	---
Knowledge score (0–11)	Per 1-point increase	1.16	1.09–1.23	**<0.001**	1.16	1.08–1.24	**<0.001**	10.4	**<0.001**

Outcome: FP willingness (willing = 1, not willing = 0). Willingness rate: 77.0% (768/998). Sample restricted to participants aged ≥ 18 years with non-missing willingness data. Personal income (Q17): coded 1–6 for valid responses, with −3 (not applicable) recoded to 0. OR = odds ratio; CI = confidence interval; PAF = population attributable fraction; ref = reference category. Bold values indicate *p* < 0.05. The variable ‘has children’ was excluded due to collinearity with marital status (aOR = 1.42, *p* = 0.648 in preliminary models). Model diagnostics: Hosmer-Lemeshow χ^2^ = 5.20 (*p* = 0.737), AUC = 0.633, all VIF < 2.53.

Overall, 77.0% (*n* = 768) of participants in the general sample expressed willingness to use FP services, while 23.0% (*n* = 230) did not. In the unadjusted analysis, several variables were significantly associated with FP willingness. Age 30–39 years (crude OR = 1.49, 95% CI: 1.09–2.03, *p* = 0.012), married status (crude OR = 1.79, 95% CI: 1.33–2.40, *p* < 0.001), FP awareness (crude OR = 1.58, 95% CI: 1.10–2.27, *p* = 0.014), and higher knowledge score (crude OR = 1.16, 95% CI: 1.09–1.23, *p* < 0.001) were all significantly associated with willingness.

In the multivariable model, two variables emerged as statistically significant independent predictors of FP willingness:

First, marital status was the strongest independent predictor (aOR = 2.10, 95% CI: 1.28–3.44, *p* = 0.003), indicating that married individuals had more than twice the odds of expressing willingness to use FP services compared with unmarried individuals, after adjusting for all other covariates. This finding suggests that the experience of marriage, and potentially the associated engagement with reproductive planning and family-building decisions, substantially enhances receptiveness to FP. Married individuals may have a more concrete and immediate appreciation of the value of preserving reproductive options, whether for future family expansion, as a contingency against health-related threats to fertility, or as a means of securing reproductive choices in the face of changing life circumstances.

Second, knowledge score was independently associated with FP willingness (aOR = 1.16, 95% CI: 1.08–1.24, *p* < 0.001), indicating that each 1-point increase on the 11-point knowledge scale was associated with a 16% increase in the odds of willingness. While the magnitude of this effect is more modest than the effect of knowledge on FP awareness (aOR = 1.86), it nonetheless confirms that knowledge plays a role in shaping both awareness and willingness, and that educational interventions targeting knowledge improvement may yield benefits at multiple stages of the awareness-to-action continuum.

Educational attainment was not independently associated with FP willingness after multivariable adjustment (aOR = 1.09, 95% CI: 0.95–1.25, *p* = 0.198), despite its theoretical importance as a determinant of health-related attitudes. This finding suggests that the influence of formal education on FP willingness is largely mediated through knowledge and other covariates included in the model.

Several variables that were significant in the unadjusted analysis did not retain significance in the multivariable model. Gender (aOR = 0.99, *p* = 0.976), age group (30–39 years: aOR = 0.98, *p* = 0.941; 40–45 years: aOR = 0.65, *p* = 0.240), personal income (aOR = 0.95, *p* = 0.354), and FP awareness (aOR = 1.00, *p* = 0.984) were not independently associated with FP willingness after adjustment for the other predictors. The loss of significance for FP awareness in the adjusted model suggests that the bivariate association between awareness and willingness is largely explained by underlying factors such as knowledge level and marital status, rather than representing a direct independent pathway. The >45 years age category yielded an elevated but non-significant adjusted odds ratio (aOR = 4.63, 95% CI: 0.59–36.49, *p* = 0.145), which should be interpreted with caution given the small number of participants in this category (*n* = 22).

The PAF analysis identified marital status as the modifiable factor with the largest population-level impact on FP willingness (PAF = 39.7%, *p* = 0.003). Given that 59.2% of the sample was married, this result suggests that the experience and context associated with marriage contribute substantially to population-level willingness to consider FP. From a public health perspective, this finding implies that interventions aimed at increasing FP willingness among unmarried individuals-who constitute a large and potentially underserved segment of the reproductive-age population-could yield meaningful gains in overall willingness. Specifically, integrating FP-related content into premarital education programs, reproductive life planning counseling, and youth-oriented health promotion activities may help bridge the willingness gap between married and unmarried individuals.

Knowledge score demonstrated a significant but smaller PAF of 10.4% (*p* < 0.001), indicating that improvements in population-level knowledge could prevent approximately 10.4% of the current unwillingness to use FP services.

Several variables that were significant in bivariate analyses-such as age group, FP awareness, and parental status-did not retain statistical significance after adjustment, suggesting that their apparent associations with FP willingness are largely explained by underlying differences in marital status and knowledge. The pattern of independent predictors was broadly consistent with the college student analysis (Model 2, Table 8): knowledge-related factors played a central role in both populations, and marital status in the general population paralleled the influence of reproductive health behaviors (contraceptive use) among college students in driving willingness to use FP services.

## 4 Discussion

### 4.1 Summary of principal findings

This study investigated FP awareness and willingness to use FP services among college students and the general population in Henan Province, central China, employing multivariable logistic regression and population attributable fraction (PAF) estimation. To our knowledge, few studies in Chinese populations have combined these approaches to identify independent predictors and quantify their population-level impact.

The principal findings are as follows. First, FP awareness was low, with only 21.1% of college students and 25.9% of the general population reporting awareness. Second, knowledge was the single most important modifiable determinant of FP awareness (aOR = 2.22 in college students; aOR = 1.86 in the general population), with a PAF of approximately 46–63%, indicating that knowledge-targeted interventions could prevent a substantial proportion of the current awareness deficit. Third, predictors of FP willingness were more multifactorial and differed across samples: gender, medical major, and contraceptive use were key predictors among college students, whereas marital status and knowledge score predominated in the general population. Fourth, the pattern of independent predictors was broadly consistent across population groups, supporting unified intervention strategies. Fifth, two novel knowledge scoring systems (13-point and 11-point scales) were developed and validated for assessing reproductive health and FP-specific knowledge.

### 4.2 Knowledge as the primary predictor of FP awareness

The most striking finding was the dominant role of knowledge in determining FP awareness. Among college students, knowledge score was the sole independent predictor after full adjustment (aOR = 2.22, 95% CI: 1.92–2.56, *p* < 0.001; PAF = 62.8%). In the general population, knowledge remained the strongest predictor (aOR = 1.86, 95% CI: 1.70–2.05, *p* < 0.001; PAF ≈ 46%). These findings indicate that knowledge is not merely one correlate among several, but the predominant predictor of FP awareness.

The magnitude of this effect is noteworthy. While studies in high-income countries have identified educational attainment and health literacy as correlates of reproductive health awareness (30–32), few have quantified the independent contribution of knowledge using multivariable regression, and none have estimated the PAF for FP awareness (30, 31, 33). Our finding that knowledge alone accounts for 46% of population-level lack of awareness provides a compelling basis for prioritizing knowledge-targeted educational interventions (34, 35).

This strong association likely reflects the fact that FP remains a specialized concept for most individuals outside medical fields (24). Unlike widely publicized topics such as contraception, FP has not been the subject of large-scale public health campaigns in China, and exposure is largely confined to formal medical education or incidental encounters (23, 36, 37). Individuals with greater factual knowledge are substantially more likely to report awareness simply because they have had more opportunities to encounter relevant information (23, 24).

The practical implication is clear: structured educational programs conveying accurate information about reproductive physiology, age-related fertility decline, the limitations of medical interventions, and available FP options have the potential to yield substantial improvements in population-level awareness (35, 38–40). The PAF estimates suggest that effectively designed and widely implemented programs could prevent the majority of the current awareness gap among young adults (33, 41).

Several alternative explanations for these associations should be considered. Reverse causation is possible: individuals who are already aware of or willing to use FP may actively seek out knowledge, rather than knowledge driving awareness. Social desirability bias may lead to overreporting of willingness in survey settings. Unmeasured confounders—including health insurance status, partner influence, media exposure, and peer effects—may account for part of the observed associations. Additionally, self-selection among online participants may have introduced systematic differences in health literacy compared with paper-based respondents.

### 4.3 Knowledge as a mediator: explaining the attenuation of demographic predictors

A particularly informative aspect of our analysis was the consistent attenuation of several demographic and behavioral variables after adjustment for knowledge score, providing evidence for a mediating role of knowledge.

Among college students, place of residence (crude OR = 0.62, *p* = 0.007 → aOR = 0.70, *p* = 0.203), medical major (crude OR = 1.70, *p* = 0.004 → aOR = 1.13, *p* = 0.586), premarital sexual activity (crude OR = 2.29, *p* < 0.001 → aOR = 1.36, *p* = 0.374), and contraceptive use (crude OR = 1.96, *p* = 0.037 → aOR = 1.25, *p* = 0.570) all lost significance after knowledge adjustment. This pattern suggests that well-documented urban–rural disparities and the advantage of medical education operate primarily through differential knowledge rather than through direct effects of living environment or academic discipline (23, 42, 43).

This finding is practically encouraging because it implies that these disparities are modifiable through knowledge-targeted interventions. If knowledge mediates the effects of residence and academic major, providing equivalent educational content to rural and non-medical students should reduce or eliminate these disparities-a more actionable strategy than attempting to change residential patterns or specialization choices (34, 37, 38, 41, 43).

In the general population, a similar mediation pattern was observed: educational attainment (crude OR = 1.31, *p* < 0.001 → aOR = 0.93, *p* = 0.363) and gender (crude OR = 1.42, *p* = 0.032 → aOR = 1.13, *p* = 0.545) lost significance after adjustment, while knowledge score and medical background retained independent effects. This consistency strengthens the conclusion that knowledge is the primary pathway through which sociodemographic factors influence FP awareness (44, 45).

### 4.4 The independent contribution of medical education

While knowledge was the dominant predictor, medical educational background retained a significant independent association with FP awareness in the general population (aOR = 1.83, 95% CI: 1.22–2.75, *p* = 0.003), even after controlling for knowledge score. This suggests that medical education confers an awareness advantage extending beyond factual knowledge captured by our scoring system, possibly through ambient awareness arising from clinical exposure, professional networks, and continuing education programs (46, 47).

Similarly, medical background independently predicted FP willingness among college students (aOR = 1.62, *p* = 0.003; PAF = 24.6%), suggesting influences through pathways distinct from knowledge alone-potentially including greater comfort with medical procedures, familiarity with cryopreservation technologies, and normalization of reproductive technology use (46, 48).

These findings imply that integrating FP content into general education should aim to replicate not only factual knowledge but also contextual exposure. This could include case-based learning modules, guest lectures by reproductive medicine specialists, or facility visits that enhance both knowledge and attitudinal familiarity (46, 48).

### 4.5 Contraceptive use and FP willingness: a novel association

Among the most novel findings was the strong independent association between contraceptive use and FP willingness among college students (aOR = 2.06, 95% CI: 1.30–3.27, *p* = 0.002; PAF = 48.2%). To the best of our knowledge, no previous study has identified or quantified this association.

This finding suggests the existence of a broader construct of “reproductive health agency”-a proactive orientation toward managing one’s reproductive life that encompasses both contraceptive use and openness to FP (49). The high prevalence of contraceptive use (87.6%) amplifies its population-level impact. Practically, existing contraceptive education programs represent an immediately available platform for integrating FP information, leveraging established infrastructure to promote FP awareness among already-engaged young adults at minimal incremental cost (35).

This association is consistent with the Health Belief Model and Theory of Planned Behavior, suggesting that individuals who have demonstrated willingness to take action regarding reproductive health may be predisposed to consider FP as another form of reproductive risk management. FP messages framed in terms of reproductive planning may therefore resonate strongly with contraceptive users (35, 49, 50).

### 4.6 Marital status and reproductive life experience

In the general population, marital status was the strongest independent predictor of FP willingness (aOR = 2.10, 95% CI: 1.28–3.44, *p* = 0.003; PAF = 39.7%). Married individuals had more than twice the odds of expressing willingness compared with unmarried individuals, even after controlling for age, education, income, knowledge, and FP awareness.

This finding supports the hypothesis that direct engagement with reproductive planning and family-building decisions-closely associated with marriage in the Chinese sociocultural context-enhances appreciation of FP’s value (51). The large PAF indicates that the willingness gap between married and unmarried individuals accounts for a substantial proportion of overall variation.

In the Chinese cultural context, discussions about fertility have traditionally been associated with marriage and considered inappropriate for unmarried individuals. This cultural norm may contribute to lower FP willingness among unmarried participants (52, 53). Culturally sensitive, stigma-free reproductive health education that normalizes early engagement with fertility planning regardless of marital status could help narrow this gap and ensure individuals make informed choices before age-related fertility decline becomes pressing (35). Our finding extends prior literature by quantifying this association through PAF analysis and demonstrating its robustness to confounding adjustment.

### 4.7 Gender differences

Among college students, male students were significantly more willing to consider FP than female students (aOR for female = 0.68, 95% CI: 0.49–0.95, *p* = 0.024)-a finding that is counterintuitive given that women bear the primary biological burden of fertility decline. Notably, this gender effect was only borderline in the unadjusted analysis (crude OR = 0.74, *p* = 0.063), reaching statistical significance only after multivariable adjustment, which suggests that confounding by other variables partially masked the independent gender association in crude analyses.

Several explanations may account for this difference. First, the technical simplicity of sperm cryopreservation compared with oocyte cryopreservation may influence gender-specific perceptions; female students may associate FP with the more complex, costly, and physically demanding process of oocyte retrieval (54, 55). Second, evolving attitudes toward motherhood among educated young Chinese women may play a role: female students were substantially more likely to accept childlessness (36.5% vs. 21.9%), suggesting that some young women increasingly question traditional expectations regarding biological parenthood (56). Third, male students may express willingness as a general principle without fully appreciating the differential procedural burden on women (54, 57).

These patterns have implications for clinical counseling. Communication strategies should address gender-specific concerns: providing balanced information about oocyte cryopreservation safety and efficacy for women, and ensuring informed engagement with broader reproductive implications for men (54, 57).

### 4.8 Consistency across population groups

The pattern of independent predictors was broadly consistent across the college student and general population analyses. In both populations, knowledge score was the strongest predictor of FP awareness, with comparable effect sizes (aOR = 2.22 in college students vs. 1.86 in the general population), and medical background was positively associated with both awareness and willingness. Demographic variables such as gender, age, and educational attainment did not retain significance after adjustment for knowledge in either population, reinforcing the central role of knowledge as a mediator.

For FP willingness, although the specific independent predictors differed between the two populations-gender, medical major, and contraceptive use among college students versus marital status and knowledge score in the general population-the underlying pattern was coherent (58, 59). In both groups, factors reflecting engagement with reproductive health (contraceptive use among students; marriage-related reproductive planning in the general population) were key predictors of willingness, while factual knowledge contributed to both awareness and willingness across populations (35).

These broadly consistent patterns provide support for unified, population-wide educational strategies centered on knowledge improvement (58, 59), while also indicating that delivery channels and messaging may benefit from tailoring to the specific life circumstances and reproductive health engagement patterns of different population segments (34).

### 4.9 Methodological contributions

The two novel knowledge scoring systems address an important methodological gap. Previous studies typically assessed FP knowledge using single items or unstructured questions without standardized instruments (13, 60, 61). Our scoring systems integrate knowledge across multiple domains, demonstrate strong associations with outcomes, employ a simple structure facilitating administration in diverse settings, and can serve as outcome measures for evaluating educational interventions. Future research should further evaluate their psychometric properties, including test–retest reliability, internal consistency, and sensitivity to change.

### 4.10 PAF-informed prioritization framework

PAF estimation assumes a causal relationship between the exposure and outcome, an assumption that cannot be verified in a cross-sectional design. The PAF values reported here should therefore be interpreted as upper-bound estimates of the proportion of the outcome attributable to each factor, contingent on the assumption of causality and the absence of residual confounding. For FP willingness outcomes (prevalence >50%), sensitivity analyses using corrected risk ratios yielded substantially lower PAF estimates (Supplementary Table S1), confirming that the magnitude of population-level impact should be interpreted cautiously for common outcomes.

Based on PAF estimates, the following framework is proposed:

Priority 1 (Highest impact): Knowledge-targeted education (PAF for awareness: 46–63%; for willingness: 10.4%). Programs should address identified knowledge gaps-particularly misconceptions about age-related fertility decline timing (35.2% correct) and unrealistic expectations regarding medical interventions (42.2% holding incorrect beliefs)-through diverse delivery formats including classroom instruction, online modules, social media content, and community workshops (38).
Priority 2: Integration into existing reproductive health programs (PAF for willingness: 48.2% for contraceptive use). Adding FP content to contraceptive counseling and sexual health education leverages established infrastructure and an already-engaged audience (35).
Priority 3: Targeted engagement of unmarried individuals (PAF for willingness: 39.7% for marital status). Strategies may include incorporating FP content into premarital education, developing youth-oriented reproductive planning tools, and culturally sensitive campaigns normalizing fertility discussions regardless of marital status (42).
Priority 4: Extension of medical education content (PAF for willingness: 24.6% for medical major). Adapting medical curricula elements for general education through interdisciplinary courses and inter-professional learning opportunities.
Priority 5: Addressing urban–rural disparities. Although residence was not independently associated with outcomes after knowledge adjustment, significant bivariate urban–rural gradients (27.5% vs. 17.1%, *p* = 0.013) indicate that ensuring equitable access to educational interventions in rural communities is essential, potentially leveraging primary care, community health workers, and mobile health technologies.

### 4.11 Limitations and future directions

Several limitations should be considered. (1) Cross-sectional design: The study cannot establish causal relationships or temporal ordering between predictors and outcomes; prospective longitudinal studies are needed to confirm directionality. (2) Selection bias: Non-probability sampling via online platforms and institutional channels may over-represent educated, urban, and health-interested individuals, potentially inflating awareness and willingness estimates. (3) Self-report bias: Social desirability may inflate reported willingness; recall bias may affect knowledge items and behavioral variables such as premarital sexual activity and contraceptive use. (4) Knowledge score validation: The scoring systems were developed for this study; Cronbach’s *α* values (0.708 and 0.783) indicate acceptable but not excellent internal consistency. Test–retest reliability and sensitivity to change were not assessed; future psychometric evaluation is warranted. (5) PAF assumptions: PAF estimation assumes causal relationships and no residual confounding. OR-based PAFs overestimate population-level impact for common outcomes (prevalence >50%); sensitivity analyses using corrected risk ratios yielded substantially lower estimates (Supplementary Table S1). (6) Generalizability: The study was conducted in a single inland province; Henan’s demographic and socioeconomic characteristics may not represent coastal or first-tier cities. Multi-center replication across diverse regions is needed. (7) Additional limitations: Two related but distinct knowledge scoring systems were used, limiting direct comparability across models; the survey assessed willingness rather than actual utilization; and the brief questionnaire limited depth of information on attitudes toward specific FP methods, perceived barriers, and partner influences.

Despite these limitations, this study makes important contributions: it is among the first to apply multivariable regression with PAF analysis to FP awareness and willingness in a Chinese population, provides standardized knowledge assessment tools, and compares predictor patterns across two distinct population groups. Future research should include longitudinal cohort studies, randomized trials evaluating educational interventions, multi-center studies, qualitative exploration of underlying beliefs, and implementation science research examining optimal delivery strategies and cost-effectiveness.

## 5 Conclusion

This study demonstrated that FP awareness among college students and the general population in Henan Province is low (21.1 and 25.9%, respectively). Through multivariable logistic regression and PAF analysis, knowledge was identified as the primary modifiable factor associated with FP awareness (aOR = 1.86–2.22; PAF = 46–63%), suggesting that knowledge-targeted interventions could substantially reduce the current awareness deficit. Predictors of FP willingness were more multifactorial, with marital status (aOR = 2.10, PAF = 39.7%), contraceptive use (aOR = 2.06, PAF = 48.2%), and medical background independently contributing. OR-based PAFs represent upper-bound estimates for common outcomes; sensitivity analyses using corrected risk ratios yielded more conservative estimates (Supplementary Table S1). If confirmed in longitudinal studies, these findings support knowledge-centered educational strategies prioritizing non-medical populations, unmarried individuals, and rural residents. Randomized controlled trials evaluating such interventions are warranted to confirm the causal pathways suggested by these cross-sectional associations.

These findings provide a PAF-informed prioritization framework for public health action. The highest priority is developing structured, knowledge-centered FP educational programs addressing key misconceptions through diverse delivery formats. Integration of FP content into existing reproductive health platforms, targeted engagement of unmarried individuals, and extension of medical education content to broader populations represent additional high-impact strategies. By addressing these modifiable determinants through evidence-based, equity-oriented interventions, health systems can support informed reproductive decision-making and promote equitable access to FP-related knowledge and services.

## Data Availability

The raw data supporting the conclusions of this article will be made available by the authors, without undue reservation.
