# Supplementary material for: Predictors of fertility preservation awareness and willingness among college students and the general population in Henan, China: a cross-sectional study
Source: Front Public Health. 2026 Jun 3;14:1830907. doi: 10.3389/fpubh.2026.1830907 (PMC13272377; doi:10.3389/fpubh.2026.1830907)
Supplement: Supplementary file 1 [file Table_1.docx]

**Supplementary Table S1. Sensitivity analysis: Population attributable fractions (PAF) estimated using adjusted odds ratios (OR) versus corrected risk ratios (RR) for FP willingness models with outcome prevalence exceeding 50%**

| **Predictor** | **Model** | **Outcome prevalence (%)** | **Adjusted OR** | **PAF (%)**  **(OR-based)** | **Corrected RR** | **PAF (%)**  **(RR-based)** |
| --- | --- | --- | --- | --- | --- | --- |
| Contraceptive use | Model 2 | 65.3 | 2.06 | 48.2 | 1.34 | 22.8 |
| Medical major | Model 2 | 65.3 | 1.62 | 24.6 | 1.18 | 8.8 |
| Marital status (married) | Model 4 | 77.0 | 2.10 | 39.7 | 1.18 | 9.8 |
| Knowledge score (IQR) | Model 4 | 77.0 | 1.34 | 10.4 | 1.08 | 2.6 |

**Notes:** OR = odds ratio; RR = risk ratio; PAF = population attributable fraction; IQR = interquartile range; FP = fertility preservation. Corrected RR was calculated using the Zhang and Yu formula: RR = OR / [(1 − P₀) + (P₀ × OR)], where P₀ is the outcome prevalence in the unexposed (reference) group(30). For the knowledge score predictor, the OR represents the effect of an interquartile range (IQR) increase, and P₀ is the willingness prevalence among participants scoring at or below the first quartile. For FP awareness models (Models 1 and 3, outcome prevalence 21–26%), adjusted ORs approximate relative risks, and PAF correction was not required. The substantial reduction in PAF estimates after RR correction (50–75%) confirms that OR-based PAFs represent upper-bound estimates for common outcomes and should be interpreted cautiously.

**Supplementary Table S2. Model diagnostics for multivariable logistic regression models (Models 1–4)**

| **Model** | **Outcome** | **n** | **Predictors** | **H-L χ²** | **df** | **H-L p** | **AUC** | **VIF range** |
| --- | --- | --- | --- | --- | --- | --- | --- | --- |
| Model 1 | College FP Awareness | 758 | 11 | 162.21 | 8 | <0.001 | 0.844 | 1.04–1.72 |
| Model 2 | College FP Willingness | 758 | 12 | 7.09 | 8 | 0.527 | 0.635 | 1.04–1.72 |
| Model 3 | General FP Awareness | 1001 | 8 | 96 | 8 | <0.001 | 0.838 | 1.02–1.49 |
| Model 4 | General FP Willingness | 998 | 9 | 5.2 | 8 | 0.737 | 0.633 | 1.02–2.53 |

**Notes:** H-L = Hosmer-Lemeshow goodness-of-fit test; AUC = area under the receiver operating characteristic curve; VIF = variance inflation factor.

The Hosmer-Lemeshow test evaluates calibration (agreement between predicted probabilities and observed proportions). A non-significant result (p > 0.05) indicates adequate fit. The significant H-L statistics for Models 1 and 3 likely reflect the known sensitivity of this test to large sample sizes (n > 500) and the presence of a strong continuous predictor (knowledge score) generating a wide range of predicted probabilities. AUC values of 0.844 and 0.838 for these models indicate excellent discriminative ability despite the significant H-L test. AUC interpretation: 0.5 = no discrimination, 0.6–0.7 = poor, 0.7–0.8 = acceptable, 0.8–0.9 = excellent, > 0.9 = outstanding. All VIF values < 5 indicate no problematic multicollinearity.

**Supplementary Table S3. Item analysis for knowledge scoring systems: endorsement rates, corrected item-total correlations, and Cronbach’s α if item deleted**

| **Scale** | **Item** | **Endorsed n (%)** | **Item-total r** | **α if item deleted** |
| --- | --- | --- | --- | --- |
| 13-pt | Q16 Fertility concept | 265 (35) | 0.242 | 0.705 |
| 13-pt | Q19 Medical restoration (reverse) | 438 (57.8) | 0.048 | 0.735 |
| 13-pt | Q21 Infertility trend | 454 (59.9) | 0.18 | 0.715 |
| 13-pt | Q20a Cancer treatment | 593 (78.2) | 0.469 | 0.672 |
| 13-pt | Q20b Autoimmune disease | 618 (81.5) | 0.429 | 0.678 |
| 13-pt | Q20c Advanced age | 715 (94.3) | 0.304 | 0.697 |
| 13-pt | Q20d Environmental pollution | 580 (76.5) | 0.48 | 0.67 |
| 13-pt | Q20e Unhealthy diet | 648 (85.5) | 0.433 | 0.679 |
| 13-pt | Q20f High-risk occupation | 601 (79.3) | 0.47 | 0.672 |
| 13-pt | Q20g Reproductive diseases | 671 (88.5) | 0.381 | 0.687 |
| 13-pt | Q23 FP awareness | 160 (21.1) | 0.351 | 0.688 |
| 13-pt | Q24 Why FP | 174 (23) | 0.334 | 0.69 |
| 13-pt | Q25 FP methods | 117 (15.4) | 0.379 | 0.685 |
| 11-pt | Q20 Fertility concept | 528 (52.7) | 0.331 | 0.782 |
| 11-pt | Q21a Cancer treatment | 795 (79.3) | 0.502 | 0.759 |
| 11-pt | Q21b Autoimmune disease | 857 (85.5) | 0.425 | 0.768 |
| 11-pt | Q21c Advanced age | 911 (90.9) | 0.332 | 0.777 |
| 11-pt | Q21d Environmental pollution | 851 (84.9) | 0.415 | 0.769 |
| 11-pt | Q21e Unhealthy diet | 887 (88.5) | 0.339 | 0.776 |
| 11-pt | Q21f High-risk occupation | 810 (80.8) | 0.453 | 0.764 |
| 11-pt | Q23 Why FP | 251 (25) | 0.498 | 0.759 |
| 11-pt | Q24 Target population | 393 (39.2) | 0.523 | 0.756 |
| 11-pt | Q25 FP methods | 315 (31.4) | 0.512 | 0.757 |
| 11-pt | Q26 When to consult | 198 (19.8) | 0.488 | 0.76 |

**Notes:** 13-pt = 13-point college student knowledge scoring system (n = 758, 13 items, Cronbach’s α = 0.708); 11-pt = 11-point general population knowledge scoring system (n = 1,002, 11 items, Cronbach’s α = 0.783). Item-total r = corrected item-total correlation (Pearson correlation between the item and the sum of all remaining items). Values > 0.20 are generally considered acceptable for knowledge scales. The low item-total correlation for Q19 (Medical restoration, r = 0.048) reflects the reverse-scored nature of this factual item; removing this item would increase α to 0.735 but was retained for content validity (assessing understanding of medical limitations is central to FP-related knowledge). α if item deleted = Cronbach’s α for the remaining items after removing the specified item. Values lower than the overall α indicate that the item contributes positively to scale reliability.
